# Nitric oxide-targeted protein phosphorylation during human sperm capacitation

**DOI:** 10.1038/s41598-021-00494-1

**Published:** 2021-10-25

**Authors:** Florentin-Daniel Staicu, Juan Carlos Martínez-Soto, Sebastian Canovas, Carmen Matás

**Affiliations:** 1 Department of Physiology, Veterinary Faculty, University of Murcia, International Excellence Campus for Higher Education and Research (Campus Mare Nostrum), Calle Campus Universitario, 11, 30100 Murcia, Spain; 2 Institute for Biomedical Research of Murcia (IMIB), Murcia, Spain; 3 IVI-RMA Global, Murcia, Spain; 4 Department of Physiology, Nursery Faculty, University of Murcia, International Excellence Campus for Higher Education and Research (Campus Mare Nostrum), Murcia, Spain

**Keywords:** Infertility, Phosphorylation

## Abstract

Among many other molecules, nitric oxide insures the correct progress of sperm capacitation by mediating phosphorylation events. For a more comprehensive understanding of how this happens, we capacitated human spermatozoa from healthy men in the presence/absence of S-Nitrosoglutathione, a nitric oxide donor, two nitric oxide synthase inhibitors, N^G^-Nitro-l-arginine Methyl Ester Hydrochloride and Aminoguanidine Hemisulfate salt and, finally, with/without l-Arginine, the substrate for nitric oxide synthesis, and/or human follicular fluid. When analyzing the phosphorylation of protein kinase A substrates and tyrosine residues, we particularly observed how the inhibition of nitric oxide synthesis affects certain protein bands (~ 110, ~ 87, ~ 75 and ~ 62 kD) by lowering their phosphorylation degree, even when spermatozoa were incubated with l-Arginine and/or follicular fluid. Mass spectrometry analysis identified 29 proteins in these species, related to: spermatogenesis, binding to the zona pellucida, energy and metabolism, stress response, motility and structural organization, signaling and protein turnover. Significant changes in the phosphorylation degree of specific proteins could impair their biological activity and result in severe fertility-related phenotypes. These findings provide a deeper understanding of nitric oxide’s role in the capacitation process, and consequently, future studies in infertile patients should determine how nitric oxide mediates phosphorylation events in the species here described.

## Introduction

Nitric oxide (NO) is a free radical which can regulate several physiological and pathological processes in mammals^1^. The cellular rates of NO synthesis mainly depend on nitric oxide synthases’ (NOS) activity and the availability of their substrate (l-Arginine) and co-factors^2^. The presence and localization of these enzymes have been extensively studied in spermatozoa belonging to different species^3^, including humans^4,5^ although their effect on sperm capacitation has not yet been fully understood.

Consequently, various studies hypothesized and investigated NO’s involvement in the acquisition of sperm fertilizing ability. Most of the evidence supports the view that at levels exceeding physiologic concentrations, disruption of sperm function occurs, but that at low levels, NO is essential for sperm function^6^. It has been shown that, at physiologic levels, NO plays an important role in sperm capacitation^7,8^, acrosome reaction^9^ and in the maintenance of sperm motility^10^.

The development and completion of the capacitation process require post-translational modifications of different proteins, particularly phosphorylation events, the dynamics of which are species-specific^11^. NO can modulate the capacitation via protein S-nitrosylation^12^ and activation of the cyclic adenosine monophosphate/protein kinase A (cAMP/PKA) pathway^13^. The latter leads to an increase in the phosphorylation levels of sperm proteins, particularly in serine, threonine and tyrosine residues^14,15^. Moreover, it has been suggested that the S-nitrosylation of adenylyl cyclase (AC) may be a possible mechanism of action of NO^16^.

Another pathway through which NO can modulate protein phosphorylation is by activating the soluble isoform of guanylate cyclase (sGC) and, thus, increasing the intracellular concentration of cyclic guanosine monophosphate (cGMP)^17^. cGMP activates the cGMP-dependent protein kinase (PKG)^18,19^ which leads to the serine/threonine phosphorylation of proteins that promote sperm capacitation and acrosome reaction^20,21^. Interestingly, since cGMP and cAMP compete for the catalytic sites of phosphodiesterases^22,23^, an increase in the intracytoplasmic cGMP concentration may inhibit cAMP degradation via cyclic nucleotide phosphodiesterase type 3^24^. The higher cAMP levels can, then, activate PKA and protein tyrosine phosphorylation (Tyr-P).

The ability to synthesize NO has not been described only in spermatozoa, but in oocytes and cumulus cells as well^3^. In fact, NO’s presence has been detected in the follicular fluid (FF) after gonadotropin stimulation^25^. Additionally, Revelli et al.^9^ demonstrated that a protein-enriched FF solution can increase the endogenous NOS activity in human sperm, thus leading to acrosome reaction in the same cells.

For these reasons, the aim of this study was to further investigate NO’s involvement in PKA activation and phosphorylation of tyrosine residues during the in vitro capacitation of human spermatozoa and how these phosphorylation events are regulated by the presence of FF.

## Results

### Phospho-PKA substrates and Tyr-P

**Figure 1:**
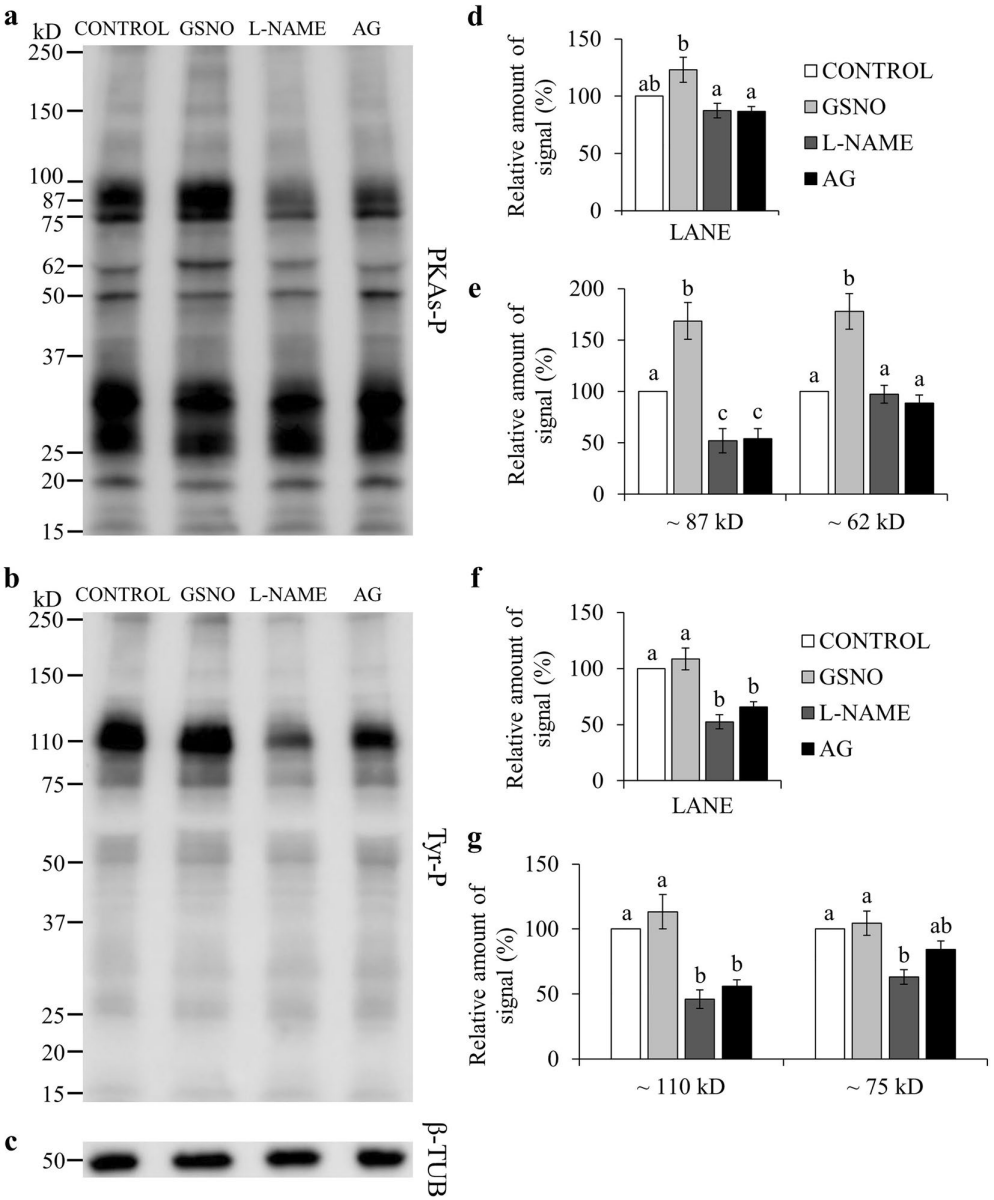
Effect of GSNO, l-NAME and AG on PKA substrates (PKAs-P) and tyrosine phosphorylation (Tyr-P). Sperm were incubated for 4 h under capacitating conditions in the absence of any treatments (CONTROL) or in the presence of GSNO, a NO donor, and l-NAME and AG (both NOS inhibitors). (**a,b**) Sperm protein extracts (n = 7) were analyzed for phosphorylation by Western blot using anti-PKAs-P or anti-Tyr-P as first antibodies, respectively. (**c**) β-tubulin (β-TUB) was used as a protein loading control. For signal quantification, each lane was normalized to its β-TUB optical density value. (**d–g**) Relative amount of signal quantified in each membrane using ImageQuant TL v8.1 software for PKAs-P and Tyr-P, respectively. Different letters (a, b, c) indicate statistically significant differences (*P* < 0.05) between groups. Images (**a**–**c**) were cropped from the corresponding blot showed in Supplementary Fig. S1 (lanes 3–6).

**Figure 2:**
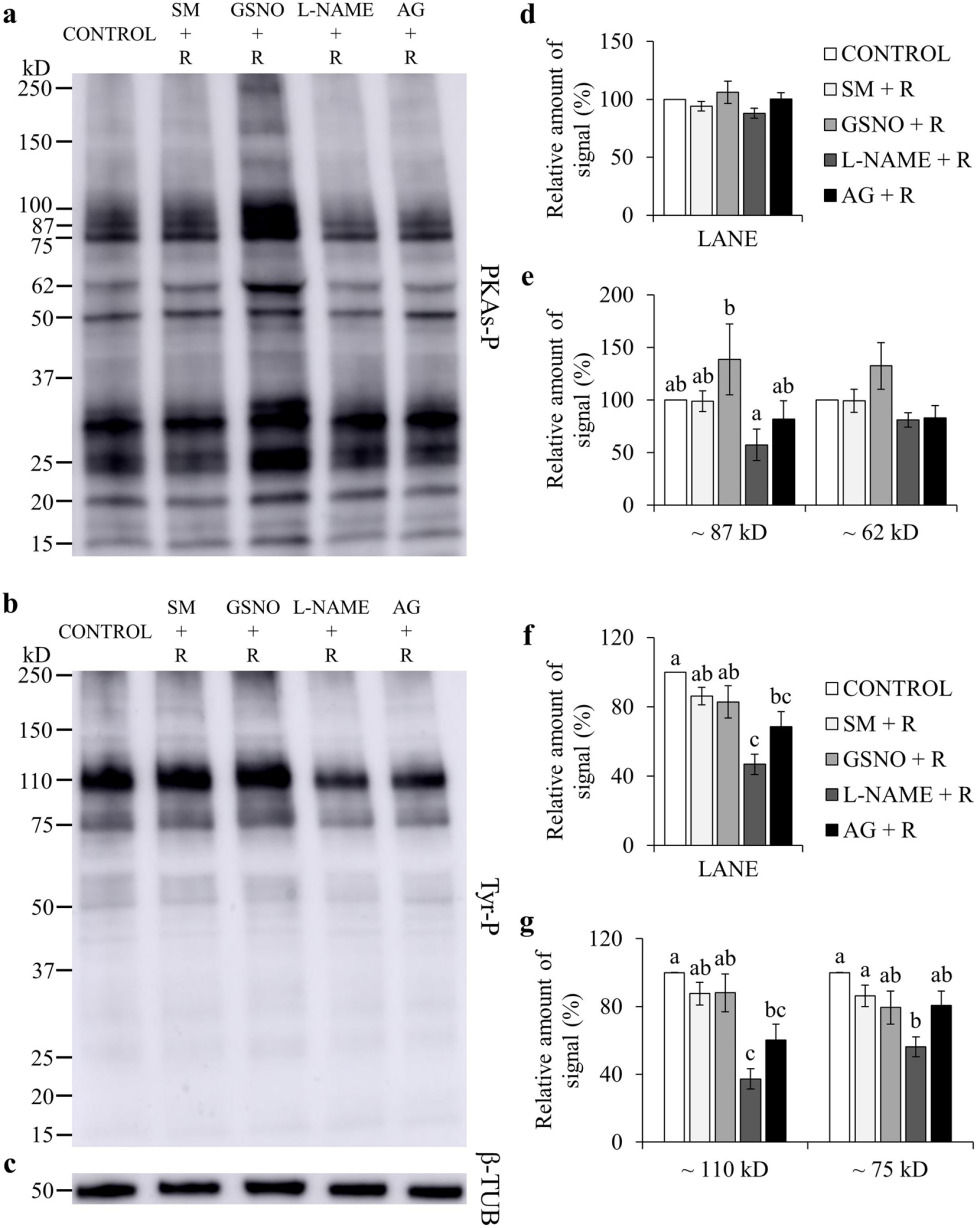
Effect of L-Arginine (R), GSNO, l-NAME and AG on PKA substrates (PKAs-P) and tyrosine phosphorylation (Tyr-P). Sperm were incubated for 4 h under capacitating conditions in the absence of any treatments (CONTROL) or in the presence of L-Arginine, the substrate for NO synthesis, GSNO, a NO donor, l-NAME and AG (both NOS inhibitors). (**a,b**) Sperm protein extracts (n = 7) were analyzed for phosphorylation by Western blot using anti-PKAs-P or anti-Tyr-P as first antibodies, respectively. (**c**) β-tubulin (β-TUB) was used as a protein loading control. For signal quantification, each lane was normalized to its β-TUB optical density value. (**d–g**) Relative amount of signal quantified in each membrane using ImageQuant TL v8.1 software for PKAs-P and Tyr-P, respectively. SM: Sperm Medium. Different letters (a, b, c) indicate statistically significant differences (*P* < 0.05) between groups. Images (**a**–**c**) were cropped from the corresponding blot showed in Supplementary Fig. S2 (lanes 3–7).

**Figure 3:**
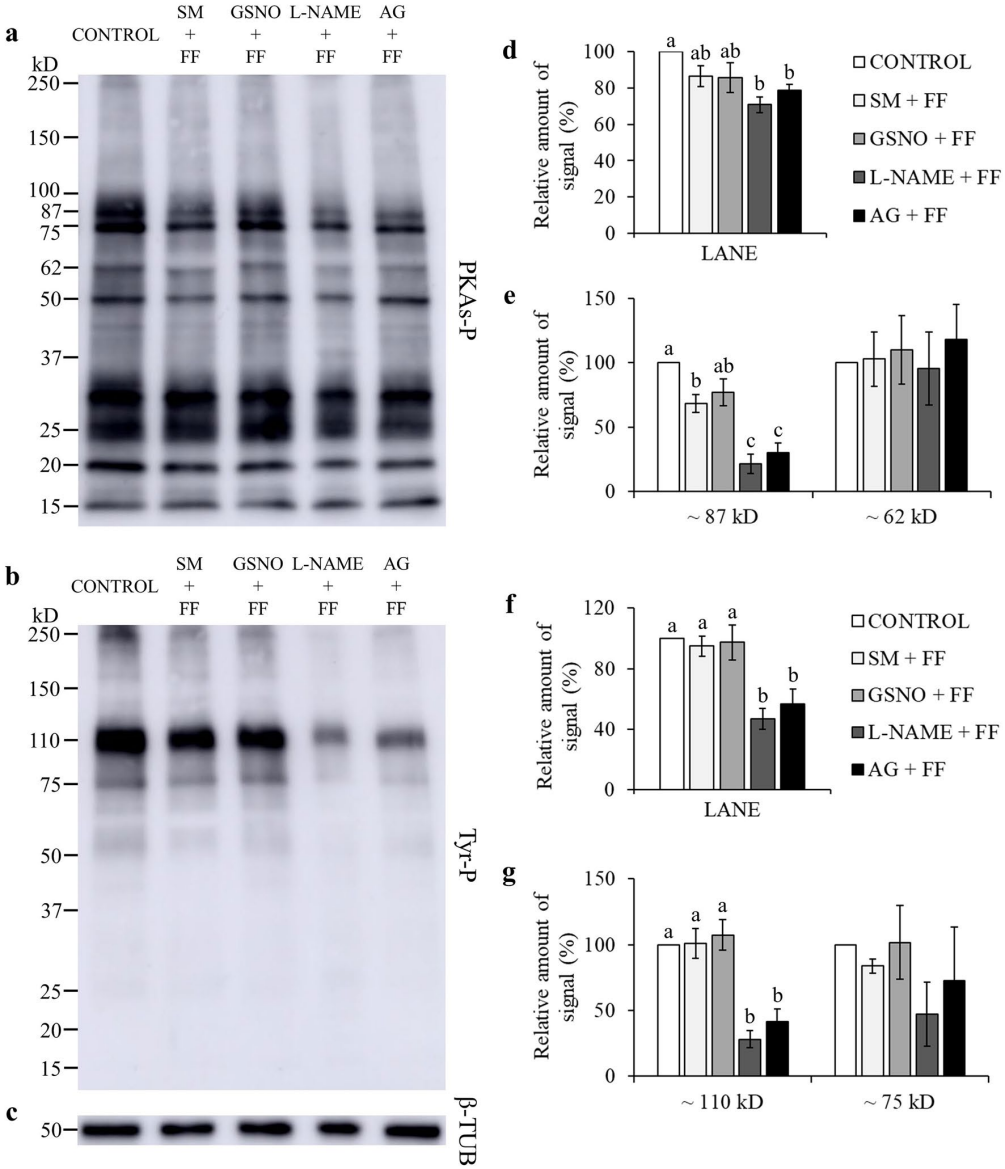
Effect of follicular fluid (FF), GSNO, l-NAME, and AG on PKA substrates (PKAs-P) and tyrosine phosphorylation (Tyr-P). Sperm were incubated for 4 h under capacitating conditions in the absence of any treatments (CONTROL) or in the presence of follicular fluid, GSNO, a NO donor, l-NAME and AG (both NOS inhibitors). (**a,b**) Sperm protein extracts (n = 7) were analyzed for phosphorylation by Western blot using anti-PKAs-P or anti-Tyr-P as first antibodies, respectively. (**c**) β-tubulin (β-TUB) was used as a protein loading control. For signal quantification, each lane was normalized to its β-TUB optical density value. (**d–g**) Relative amount of signal quantified in each membrane using ImageQuant TL v8.1 software for PKAs-P and Tyr-P, respectively. SM: Sperm Medium. Different letters (a, b, c) indicate statistically significant differences (*P* < 0.05) between groups. Images (**a**–**c**) were cropped from the corresponding blot showed in Supplementary Fig. S3 (lanes 3–7).

**Figure 4:**
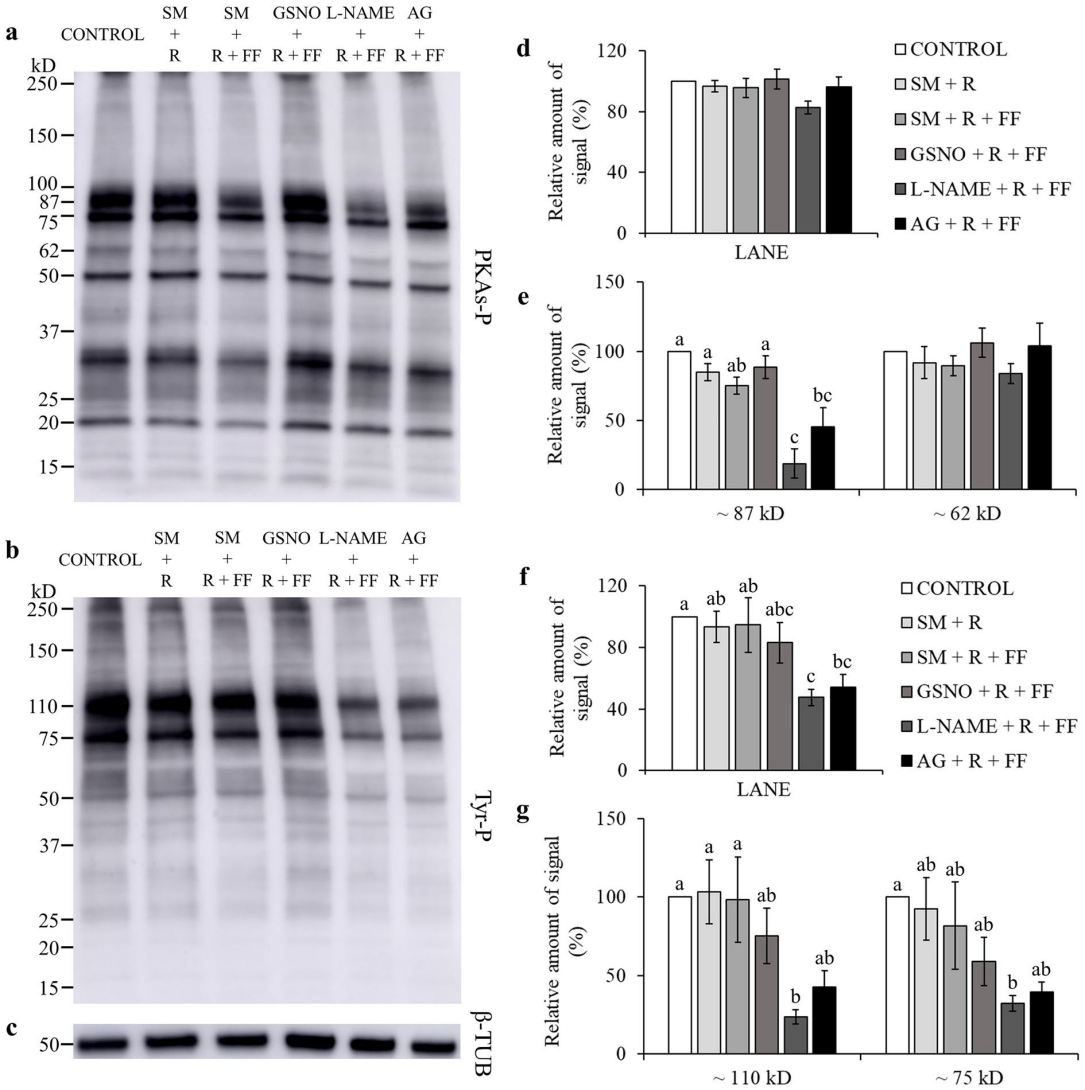
Effect of L-Arginine (R), follicular fluid (FF), GSNO, l-NAME, and AG on PKA substrates (PKAs-P) and tyrosine phosphorylation (Tyr-P). Sperm were incubated for 4 h under capacitating conditions in the absence of any treatments (CONTROL) or in the presence of L-Arginine, the substrate for NO synthesis, follicular fluid, GSNO, a NO donor, l-NAME and AG (both NOS inhibitors). (**a,b**) Sperm protein extracts (n = 7) were analyzed for phosphorylation by Western blot using anti-PKAs-P or anti-Tyr-P as first antibodies, respectively. (**c**) β-tubulin (β-TUB) was used as a protein loading control. For signal quantification, each lane was normalized to its β-TUB optical density value. (**d–g**) Relative amount of signal quantified in each membrane using ImageQuant TL v8.1 software for PKAs-P and Tyr-P, respectively. SM: Sperm Medium. Different letters (a, b, c) indicate statistically significant differences (*P* < 0.05) between groups. Images (**a**–**c**) were cropped from the corresponding blot showed in Supplementary Fig. S4 (lanes 3–8).

Many studies evidenced that for spermatozoa to achieve their fertilizing ability, a fine regulation of protein phosphorylation is required^26^. Total levels of phospho-PKA substrates were significantly lowered by l-NAME and AG, when compared to GSNO (*P* < 0.05; Fig. 1a,d; Supplementary Fig. S1). GSNO significantly increased the phosphorylation degree of two PKA substrates of approximately ~ 87 kD and ~ 62 kD, when compared to CONTROL (Fig. 1a,e; Supplementary Fig. S1). NOS inhibitors significantly lowered the phosphorylation degree of the ~ 87 kD species, compared to CONTROL and GSNO, and of the ~ 62 kD species, only when compared to GSNO (Fig. 1a,e; Supplementary Fig. S1). When adding l-Arginine, the relative amount of signal for the ~ 87 kD species was lower with l-NAME than in the GSNO group (*P* < 0.05; Fig. 2a,e; Supplementary Fig. S2). The FF supplementation decreased the phosphorylation degree of the ~ 87 kD species (Fig. 3a,e; Supplementary Fig. S3). Moreover, the addition of l-NAME and AG significantly inhibited the total levels of phospho-PKA substrates and particularly the ~ 87 kD band (Fig. 3a,d,e; Supplementary Fig. S3). During the simultaneous supplementation with l-Arginine and FF only the ~ 87 kD band was affected by the NOS inhibitors, which diminished its signal (Fig. 4a,d,e; Supplementary Fig. S4).

Total phosphorylation degree of tyrosine residues was found to be significantly lower than CONTROL and GSNO, when using NOS inhibitors (Fig. 1b,f; Supplementary Fig. S1). In the presence of l-Arginine, l-NAME’s inhibitory effect was maintained for both total and band-specific Tyr-P levels (Fig. 2b,f,g; Supplementary Fig. S2). AG decreased total Tyr-P levels and the ~ 110 kD band, compared to CONTROL, although its effect was not as noticeable as with l-NAME (Fig. 2b,f,g; Supplementary Fig. S2). When examining the FF supplementation, NOS inhibitors significantly decreased both global and the ~ 110 kD Tyr-P levels (Fig. 3b,f,g; Supplementary Fig. S3). Furthermore, during the co-incubation with l-Arginine and FF, l-NAME led to lower overall and band-specific Tyr-P levels (Fig. 4b,f,g; Supplementary Fig. S4). Under the same experimental conditions, AG also inhibited total Tyr-P levels (Fig. 4b,f; Supplementary Fig. S4).

### Mass spectrometry analysis

**Figure 5:**
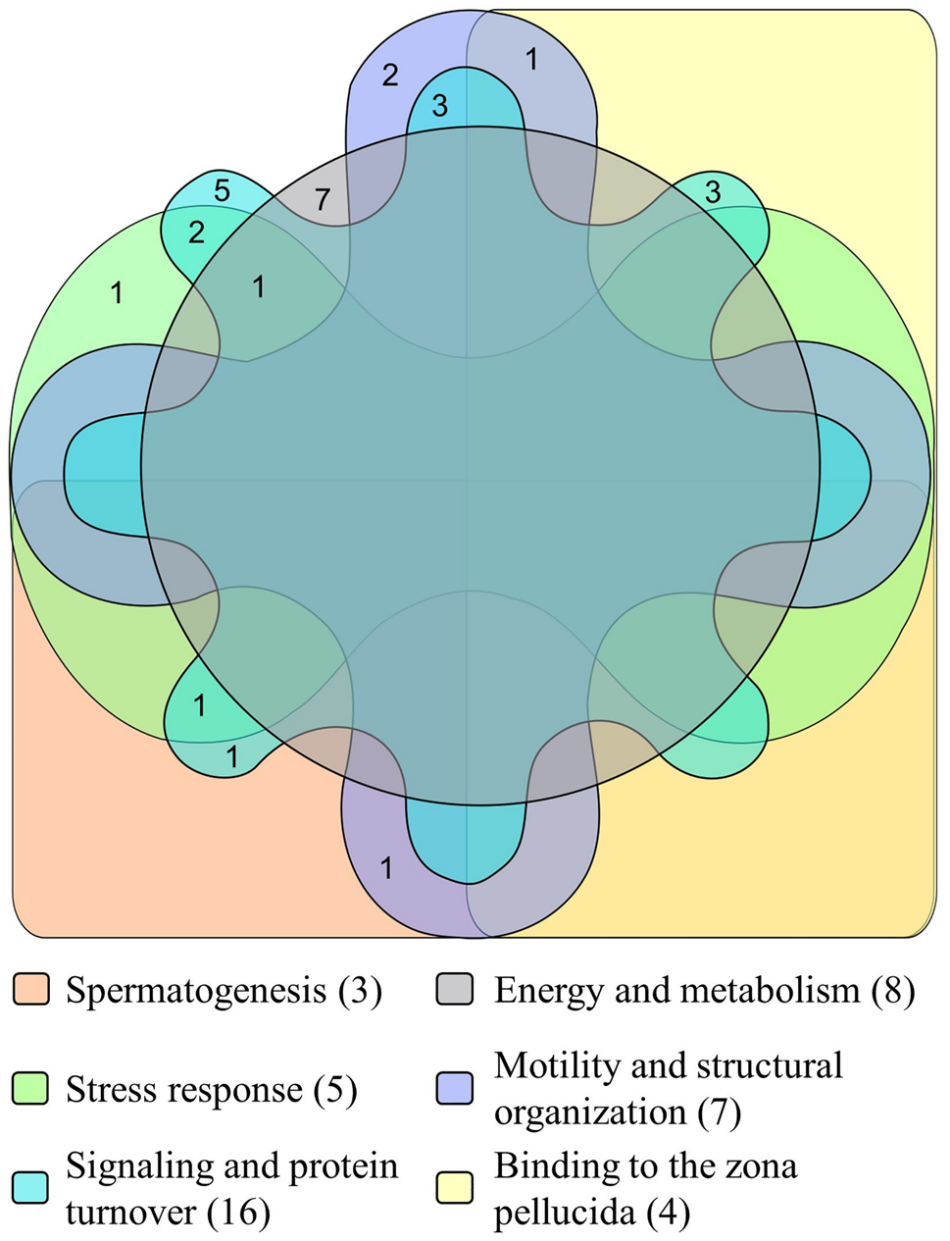
Venn diagram illustrating different functional groups for the proteins identified via HPLC-ESI-Q-TOF–MS/MS. The figure was created using the web-based tool InteractiVenn^88^.

Four protein bands were selected (~ 110, ~ 87, ~ 75 and ~ 62 kD) and a total of 29 proteins were identified after HPLC-ESI-Q-TOF–MS/MS analysis (Table 1). These proteins belong to six different functional groups (Fig. 5), as follows: (i) *spermatogenesis* (outer dense fiber of sperm tails 2, isoform CRA_c—ODF2—; heat shock 70 kDa protein 2, isoform CRA_a—HSPA2—; mitochondria-eating protein—SPATA18—), (ii) *binding to the zona pellucida* (T-complex protein 1 subunits gamma—CCT3—, alpha—TCP1—, theta—CCT8—and eta—CCT7—), (iii) *energy and metabolism* (hexokinase 1, isoform CRA_c—HK1—; neutral alpha-glucosidase AB—GANAB—; transitional endoplasmic reticulum ATPase—VCP—; ATP-dependent 6-phosphofructokinase, platelet type—PFKP—; alpha-1,4 glucan phosphorylase; glutamine—fructose-6-phosphate aminotransferase [isomerizing] 1—GFPT1—; pyruvate kinase—PKM2—; pyruvate kinase PKM—PKM—), (iv) *stress response* (epididymis luminal secretory protein 52—EL52—; heat shock protein 90 kDa alpha (Cytosolic), class B member 1, isoform CRA_a—HSP90AB1—; VCP; endoplasmic reticulum chaperone BiP—HSPA5—; HSPA2), (v) *motility and structural organization* (actinin, alpha 1, isoform CRA_a—ACTN1—; endoplasmin—HSP90B1—; A kinase (PRKA) anchor protein 4, isoform CRA_c—AKAP4—; A-kinase anchor protein 3—AKAP3—; ODF2; TCP1; WD repeat-containing protein 1—WDR1—), and (vi) *signaling and protein turnover* (26S proteasome non-ATPase regulatory subunit 2—PSMD2—; testicular secretory protein Li 63—UBE1—; HSP90AB1; HSP90B1; AKAP4; AKAP3; VCP; HSPA5; HSPA2; lactoferrin—LTF—; CCT3; CCT8; CCT7; epididymis secretory sperm binding protein—HSPD1—; chaperonin containing TCP1, subunit 6A (Zeta 1), isoform CRA_a—CCT6A—; SPATA18).

**Table 1:** List of proteins identified via HPLC-ESI-Q-TOF–MS/MS in human spermatozoa.

Band (kD)	Accession No. (UniProtKB)	Protein name	Gene name	Molecular weight (kD)	No. of peptides	Sequence coverage (%)^1^	Biological functions
** ~ 110**	A0A024QZK7	Hexokinase 1, isoform CRA_c	HK1	102.7	15	15.9	Glycolytic process; glucose homeostasis; fructose metabolism
	F5H6X6	Neutral alpha-glucosidase AB	GANAB	112.9	5	5.5	Carbohydrate metabolism
	Q13200	26S proteasome non-ATPase regulatory subunit 2	PSMD2	100.2	3	3.5	Proteasome-mediated ubiquitin-dependent protein catabolism; MAPK cascade; post-translational protein modification
	A0A024R694	Actinin, alpha 1, isoform CRA_a	ACTN1	103.1	3	3.3	Actin filament bundle assembly; actin crosslink formation
	A0A024R1A3	Testicular secretory protein Li 63	UBE1	117.8	3	3.3	Ubiquitin activating enzyme activity
** ~ 87**	K9JA46	Epididymis luminal secretory protein 52	EL52	84.7	39	49.5	Protein folding; response to cold or heat
	A0A024RD80	Heat shock protein 90 kDa alpha (Cytosolic), class B member 1, isoform CRA_a	HSP90AB1	83.3	33	42.1	Protein kinase regulator activity; regulation of proteasomal protein catabolic process; placenta development; regulation of cyclin-dependent protein kinase activity; regulation of peptidyl-serine phosphorylation
	P14625	Endoplasmin	HSP90B1	92.5	10	13.2	Actin rod assembly; regulation of apoptotic process; post-translational protein modification; regulation of phosphoprotein phosphatase activity; sequestering of calcium ions
	A0A384MQY7	A kinase (PRKA) anchor protein 4, isoform CRA_c	AKAP4	94.5	23	29.6	Protein binding and localization; sperm motility
	O75969	A-kinase anchor protein 3	AKAP3	94.7	9	12.8	Protein binding and localization; sperm motility; capacitation; acrosome reaction; blastocyst hatching; regulation of protein serine/threonine kinase signaling pathway
	P55072	Transitional endoplasmic reticulum ATPase	VCP	89.3	8	13.5	Cellular response to DNA damage stimulus and heat; flavin adenine dinucleotide catabolic process; NADH metabolic process; mitotic spindle disassembly; regulation of mitochondrial membrane potential and oxidative phosphorylation; protein folding
	Q01813	ATP-dependent 6-phosphofructokinase, platelet type	PFKP	85.6	5	7.6	Glycolysis; fructose 1,6-bisphosphate and fructose 6-phosphate metabolic process
	B4DSD8	Alpha-1,4 glucan phosphorylase	*N/A*	85.8	3	5	Carbohydrate metabolism
** ~ 75**	A0A024R8A4	Outer dense fiber of sperm tails 2, isoform CRA_c	ODF2	73.3	22	32.4	Cilium organization; spermatid development
	P11021	Endoplasmic reticulum chaperone BiP	HSPA5	72.3	21	36.8	Cellular response to cAMP, calcium ions, heat, drugs, radiation and unfolded proteins; protein ubiquitination; regulation of apoptotic process
	A0A024R6B5	Heat shock 70 kDa protein 2, isoform CRA_a	HSPA2	70.0	14	22.8	Male meiosis I; regulation of protein phosphorylation; response to cold or heat; spermatid development
	A0A161I202	Lactoferrin	LTF	78.3	9	15.6	Regulation of protein serine/threonine kinase activity; regulation of ATPase activity, membrane potential and apoptotic process
	Q06210	Glutamine–fructose-6-phosphate aminotransferase [isomerizing] 1	GFPT1	78.8	5	7	Glutamine and fructose 6-phosphate metabolism; protein N-linked glycosylation
** ~ 62**	A0A024R5Z9	Pyruvate kinase	PKM2	58.1	23	44.4	ATP biosynthesis; glucose metabolism
	P14618	Pyruvate kinase PKM	PKM	57.9	22	47	ATP biosynthesis; glucose metabolism
	P49368	T-complex protein 1 subunit gamma	CCT3	57.9	12	28.5	Binding of sperm to zona pellucida; protein folding
	P17987	T-complex protein 1 subunit alpha	TCP1	60.3	13	24.4	Binding of sperm to zona pellucida; protein folding; tubulin complex assembly
	P50990	T-complex protein 1 subunit theta	CCT8	59.6	6	13.6	Binding of sperm to zona pellucida; protein folding
	A0A024R3X4	Epididymis secretory sperm binding protein	HSPD1	61.1	6	10.2	De novo protein folding; protein refolding; mitochondrion organization; regulation of apoptotic process
	B3KN28	Phosphoacetylglucosamine mutase	*N/A*	59.9	4	6	Carbohydrate metabolism; UDP-N-acetylglucosamine biosynthetic process
	Q99832	T-complex protein 1 subunit eta	CCT7	59.3	3	5.1	Binding of sperm to zona pellucida; protein folding
	O75083	WD repeat-containing protein 1	WDR1	62.1	3	8	Actin filament depolymerization and fragmentation
	A0A024RDL1	Chaperonin containing TCP1, subunit 6A (Zeta 1), isoform CRA_a	CCT6A	58.0	3	8	Protein folding
	Q8TC71	Mitochondria-eating protein	SPATA18	61.1	2	4.4	Mitochondrial protein catabolism; spermatogenesis

^1^Percentage of the protein sequence covered by identified peptides.

### Protein-proteins interactome and knockout phenotypes related to sperm proteins affected by phosphorylation

**Figure 6:**
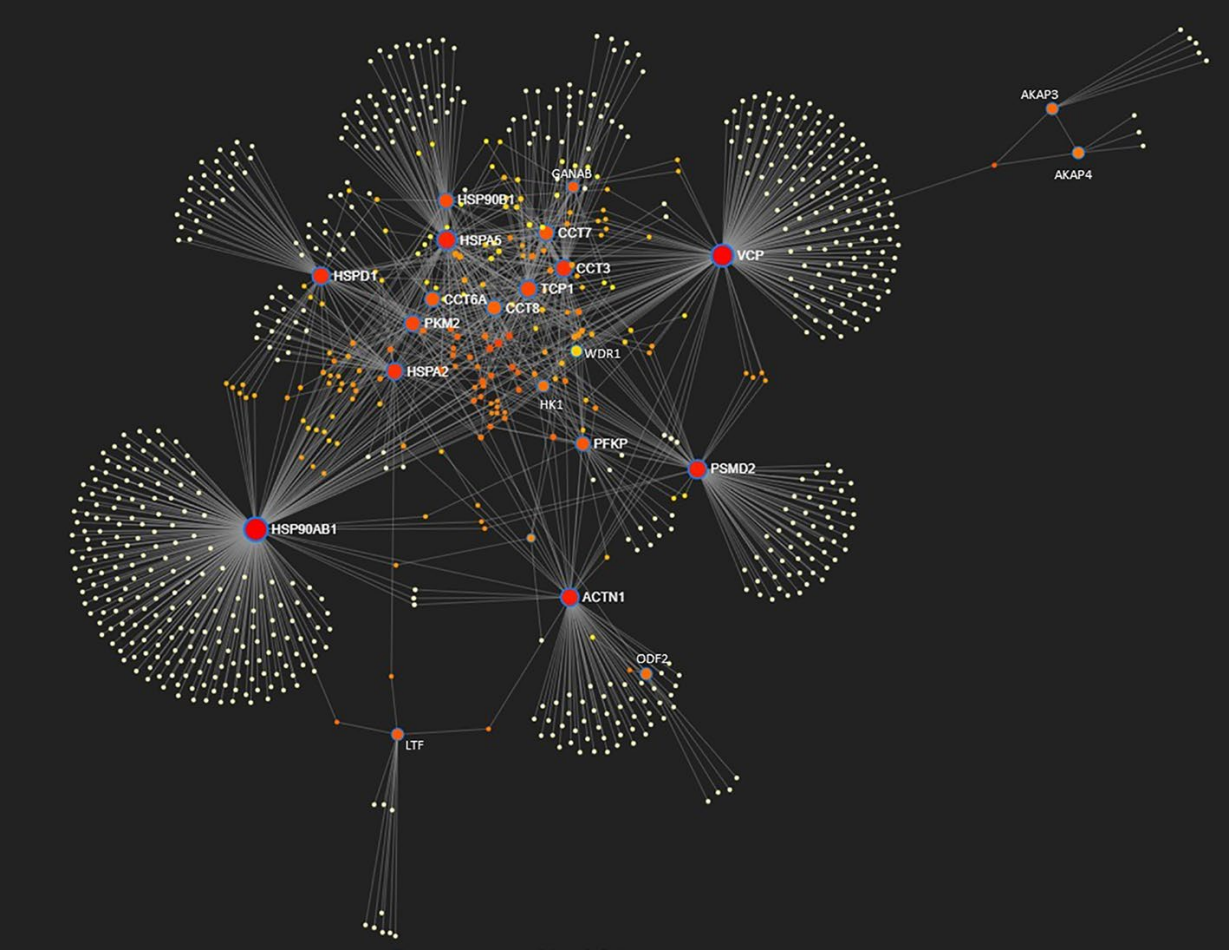
Protein–protein interactions network produced by NetworkAnalyst 3.0. Significant factors (seeds) are highlighted with a blue circle. The size of the nodes is based on their degree (connectivity) values, with a big size for large degree values. The color of the nodes is proportional to their betweenness centrality values, starting from red for higher values.

For the 29 human sperm proteins identified in Table 1, a protein–protein interactome was constructed using NetworkAnalyst^27^. One first-order network was produced with 22 factors identified as significant factors (seeds); 774 nodes and 1155 edges were reported for this network. According to connectivity values (Supplementary Table S5), the top ten high degree nodes (hubs) were: HSP90AB1, VCP, HSPA5, PSMD2, ACTN1, HSPD1, CCT3, TCP1, HSPA2 and PKM2 (Fig. 6).

Using the same list of proteins, we searched for mammalian knockout phenotypes related to fertility and 12 genes were identified (Table 2). From these, nine genes were present in the ~ 87 kD or ~ 62 kD bands, whose phosphorylation degree was affected by GSNO or FF. Knockouts of EL52, AKAP4, ODF2 and HSPA2 showed phenotypic alterations in sperm physiology and morphology, which resulted in male infertility. In addition, knockouts in other factors, such as HSP90AB1, HSP90B1, VCP, PKM2, CCT3, HSPD1 and WDR1, showed embryonic lethality at different stages (Table 2).

**Table 2:** Fertility-related knockout phenotypes in mammals.

Protein name	Gene name	Knockout phenotype
Epididymis luminal secretory protein 52	EL52	Male infertility; small testis; testicular atrophy; arrest of spermatogenesis; azoospermia; abnormal male meiosis; arrest of male meiosis; abnormal male germ cell apoptosis
Heat shock protein 90 kDa alpha (Cytosolic), class B member 1, isoform CRA_a	HSP90AB1	Abnormal placenta vasculature; abnormal trophoblast layer morphology; embryo tissue necrosis
Endoplasmin	HSP90B1	Decreased embryo size; abnormal extraembryonic endoderm formation; embryonic growth arrest; abnormal embryonic-extraembryonic morphology; disorganized embryonic tissue; embryonic lethality between implantation and placentation
A kinase (PRKA) anchor protein 4, isoform CRA_c	AKAP4	Male infertility; abnormal sperm physiology; abnormal sperm flagellum morphology; coiled sperm flagellum; short sperm flagellum; reduced hyperactivated sperm motility; abnormal sperm mitochondrial sheath morphology; abnormal sperm principal piece morphology
Transitional endoplasmic reticulum ATPase	VCP	Embryonic lethality before implantation
Outer dense fiber of sperm tails 2, isoform CRA_c	ODF2	Male infertility; asthenozoospermia; oligozoospermia; impaired acrosome reaction; detached sperm flagellum; coiled sperm flagellum; abnormal sperm midpiece morphology; abnormal sperm mitochondrial sheath morphology
Endoplasmic reticulum chaperone BiP	HSPA5	Failure of blastocyst to hatch from the zona pellucida; abnormal blastocyst morphology; abnormal inner cell mass apoptosis; embryonic lethality at implantation; embryonic lethality prior to organogenesis
Heat shock 70 kDa protein 2, isoform CRA_a	HSPA2	Arrest of spermatogenesis; male infertility; decreased testis weight; abnormal male meiosis
Pyruvate kinase	PKM2	Embryonic lethality at implantation
T-complex protein 1 subunit gamma	CCT3	Embryonic lethality between somite formation and embryo turning
Epididymis secretory sperm binding protein	HSPD1	Decreased embryo size; embryonic lethality between implantation and placentation
WD repeat-containing protein 1	WDR1	Embryonic lethality during organogenesis; embryonic lethality between implantation and somite formation; embryonic lethality prior to organogenesis

The search was performed using the MGI Batch Query search tool in the Mouse Genome Informatics database (http://www.informatics.jax.org/).

## Discussion

For the gamete interaction to be successful during fertilization, spermatozoa need to undergo structural and functional changes, which are globally known as capacitation^28^ and they are, in part, mediated by NO^29^. NO emerged as one the messengers involved in regulating protein phosphorylation levels, in particular on serine, threonine and tyrosine residues^14,30,31^. Phosphorylation acts as a switch to turn protein activity on or off (reviewed by^32^). Modifications in the phosphorylation degree of key proteins related to fertilization could result in lack or alteration of their biological activity, and consequently in unsuccessful reproductive function. Therefore, the analysis of the sperm protein phosphorylation pattern, and how in vitro culture conditions can modulate it, is critical for the success of the Assisted Reproductive Technologies. In the present paper, we provide new evidence on how NO regulates this aspect.

Our results showed an increase in the phosphorylation degree of two PKA substrates, namely ~ 87 kD and ~ 62 kD, when using the NO donor called GSNO. Similarly, Rahman et al.^31^ reported that sodium nitroprusside, another NO-releasing agent, increased the levels of different PKA substrate species in mouse spermatozoa. However, we did not observe any effect of GSNO on tyrosine phosphorylation. This contrasts previous studies, where the authors described an increase in Tyr-P when using NO donors^14^ in specific human^30^ and mouse sperm proteins^31^. We hypothesize that other molecules might have been responsible for Tyr-P in our experiment, therefore, concealing any effect of GSNO. In fact, it has been shown that the superoxide anion and hydrogen peroxide, which are endogenously generated by spermatozoa, can also induce Tyr-P and capacitation^33–35^.

When we inhibited NO synthesis, especially with l-NAME, the phosphorylation degree of PKA substrates and tyrosine residues decreased significantly. Our observations agree with other reports, in which NOS inhibitors suppressed Tyr-P in human^14,30^ and buffalo^36^ sperm proteins. Interestingly, the phosphorylation level of the ~ 87 kD band was lowered by the NOS inhibitors, even when compared to the CONTROL group, which confirms that NO synthesis takes place under our experimental conditions. This result clearly suggests that NO mediates the phosphorylation of this species and this might be an important step that should be managed under in vitro conditions to promote human sperm capacitation. Furthermore, the NO-triggered phosphorylation of this species by PKA, on serine and threonine residues, might be required prior to its phosphorylation on tyrosine residues^15^. This hypothesis should be tested in future studies.

Previous studies found that l-Arginine, a NO precursor, is a capacitating agent and l-NAME reduces the l-Arginine -induced capacitation in buffalo and bovine spermatozoa^36,37^. Moreover, Thundathil et al.^30^ observed that l-Arginine caused an increase in a threonine-glutamine-tyrosine motif in two different human sperm proteins and this effect was prevented by l-NAME. Similar results were described by Roy and Atreja^36^ when analyzing Tyr-P. In contrast to these works, we did not find any differences in the total levels of phospho-PKA substrates and Tyr-P, when adding l-Arginine to the capacitation medium, which is similar to our observations in the presence of GSNO. The reason for this could be that present levels of NO may have been sufficient to induce sperm protein phosphorylation^38^. However, the simultaneous presence of l-Arginine and l-NAME did not alter l-NAME’s inhibitory effect on total Tyr-P and phosphotyrosine levels of the ~ 110 kD and ~ 75 kD bands. This could be explained by the fact that l-NAME is a potent inhibitor of constitutive isoforms^39,40^. We also observed that when l-Arginine was used, the effect of AG on Tyr-P was not as marked as with l-NAME, which could be attributed to AG’s ability to selectively inhibit one of three NOS isoforms, responsible for NO production^41^. Therefore, we might assume that l-Arginine might have partially reverted AG’s effect, compared to l-NAME.

Since in vitro fertilization techniques make possible the recovery of human FF, several studies aimed to determine its effects on human sperm function (summarized by^42^). Our observations agree with a previous work in which no modification in the Tyr-P signal of human sperm was observed in the presence of FF, even though this fluid was used at a higher concentration^42^. Interestingly, the ~ 87 kD species exhibited a lower degree of phosphorylation in the presence of FF. Munuce et al.^42^ reported a reduction in the number of human spermatozoa bound to hemizonas after being exposed to FF, which was associated with a significant loss in mannose-binding sites in these cells. The latter have been proposed as zona pellucida recognition molecules^43^. Certain glycoproteins in the FF seem to inhibit sperm binding to the zona pellucida^44,45^, possibly by masking the mannose residues on sperm surface^42,46^. For these reasons, the phosphorylation to a lesser extent of the ~ 87 kD band, observed in the presence of FF in our study, might be important for the correct distribution of mannose-binding sites in spermatozoa. The FF supplementation did not revert the NOS inhibitors’ effect in our study, which lowered, also in this case, the levels of phospho-PKA substrates and Tyr-P. Furthermore, we observed that during the co-incubation with l-Arginine and FF, spermatozoa continued to display low Tyr-P levels in the presence of NOS inhibitors, particularly with l-NAME. However, although global phosphorylation levels of PKA substrates did not exhibit changes under these conditions, the ~ 87 kD band presented a low phosphorylation degree, which clearly suggests its importance during sperm capacitation.

Our data confirm that NO is a very important mediator of phosphorylation events during capacitation, particularly, the presence/absence of NO seemed to affect certain WB bands more than others. The proteins identified in these bands belongs to six groups depending on their function/s. Even though all these proteins could be important for reproduction, we will discuss mainly the role of the proteins with a critical function during fertilization (including binding to the zona pellucida, motility and structural organization, energy and metabolism, stress response) as they are the events that could be influenced by the capacitation environment.

CCT3, identified in the ~ 62 kD band, whose phosphorylation degree increased in the presence of GSNO and acting as a hub in the protein network, is a known component of the chaperonin-containing TCP-1 complex^47^. This complex has a profound influence in remodeling the sperm surface to become competent for zona pellucida binding and acrosome reaction^48^. Interestingly, CCT3 is weakly labeled in non-capacitated human spermatozoa and the staining increases in the peri-acrosomal region and flagellum following capacitation^47^, which reinforces its relevance for sperm-egg interactions. Moreover, downregulation of CCT3 has been reported in asthenozoospermic testicular cancer patients^49^.

Other network hubs concern either the regulation of sperm movement or the actin filament assembly/depolymerization. AKAP4, HSP90B1 and WDR1 were identified in the ~ 87 and ~ 62 kD bands respectively, whose phosphorylation degree was affected by the either the NO donor (GSNO) or FF. Specifically, AKAP4 was described in the fibrous sheath of the sperm flagellum, where it can bind phosphodiesterase isoforms or the regulatory subunit of PKA (summarized by^12^), and consequently, regulate sperm motility^50^. Furthermore, it was demonstrated that it is subjected to S-nitrosylation, which is confirmed by the presence of an S-nitrosylation motif in its primary sequence^12^. Furthermore, the targeted disruption of the AKAP gene causes male infertility in mice, with defects in sperm flagellum and motility^51^. Both HSP90B1and WDR1 seem to play a role in the actin filament turnover once their phosphorylation is triggered by NO. In particular, the former is a calcium-binding glycoprotein engaged in the assembly of multimeric protein complexes^52,53^, whereas the latter is an actin interacting protein involved in the depolymerization of actin filaments^54^. The turnover of these filaments is of great importance for sperm physiology, in particular, during the acrosome reaction^55^, and depends on PKA activation, targeted-protein phosphorylation and intracellular calcium concentration^56^. In addition, HSP90B1and WDR1 knockouts lead to fertility-related phenotypes^57–59^.

Another functional group of proteins in our study concerns energy production, which is a key factor supporting sperm motility. Spermatozoa synthesize ATP from different substrates (glucose, pyruvate and lactate), but the preferred metabolic pathway for ATP production during sperm capacitation depends on the species^60,61^. In this context, two other significant hubs showed a different phosphorylation pattern in the presence/absence of NO, namely VCP and PKM2. A previous study reported that VCP undergoes Tyr-P during capacitation followed by a change in its subcellular localization (from the neck to the anterior region of the head), suggesting a role during acrosome reaction^62^. Additionally, VCP mutations were associated with decreased mitochondrial membrane potential and increased mitochondrial oxygen consumption, which led to lower ATP levels^63^. On the other hand, PKM2 is involved in the generation of ATP during glycolysis by catalyzing the transphosphorylation from phosphoenolpyruvate to ADP^64^. Recent data suggests that PKM2 could be a marker for predicting human sperm freezability, since poor freezability ejaculates have a lower expression of this protein^65^.

The phosphorylation degree of some heat shock proteins, belonging to the 90 kD and 70kD families, was also modulated by NO in our study, and specifically, four important hubs were identified: EL52, HSP90AB1, HSPA5 and HSPA2. These chaperones regulate protein folding and apoptosis, particularly, they can either induce or inhibit the latter process during spermatogenesis,^66^. Moreover, evidence suggests that heat shock proteins commonly undergo S-nitrosylation in human sperm and can be tyrosine phosphorylated during capacitation^12^. Interestingly, a low expression of HSPA2 correlates with fewer zona pellucida binding sites in human spermatozoa^67^, which was shown to cause male infertility by impairing the interaction between gametes in vitro^68^. In addition, the expression of HSPA5 was reported in sperm^69,70^ and oviductal epithelial cells^71^, suggesting its role in the reservoir formation, prior to ovulation^72^. This clearly shows the importance of NO-mediated post-translational changes (phosphorylation and S-nitrosylation) in these chaperones to ensure proper gamete interaction and fertilization.

Finally, a considerable amount of the proteins identified in our WB bands are involved in signaling pathways, which is not surprising since their fine-tuning is essential for sperm to acquire their fertilizing ability. The cAMP/PKA, cGMP/PKG and the extracellular-regulated kinase pathways trigger highly compartmentalized phosphorylation events of proteins which govern sperm motility, viability, hyperactivation and acrosome reaction^11,73^.

## Conclusions

The present work underlines the involvement of NO in the progression of phosphorylation events during human sperm capacitation. We evidenced that specific proteins seem most likely to be targeted for serine, threonine and tyrosine phosphorylation, downstream of NO-mediated signaling. Understanding whether their phosphorylation is similarly regulated or not by NO in infertile men might improve the current knowledge regarding the etiology of infertility, particularly when its causes are not clear.

## Materials and methods

### Experimental design

**Figure 7:**
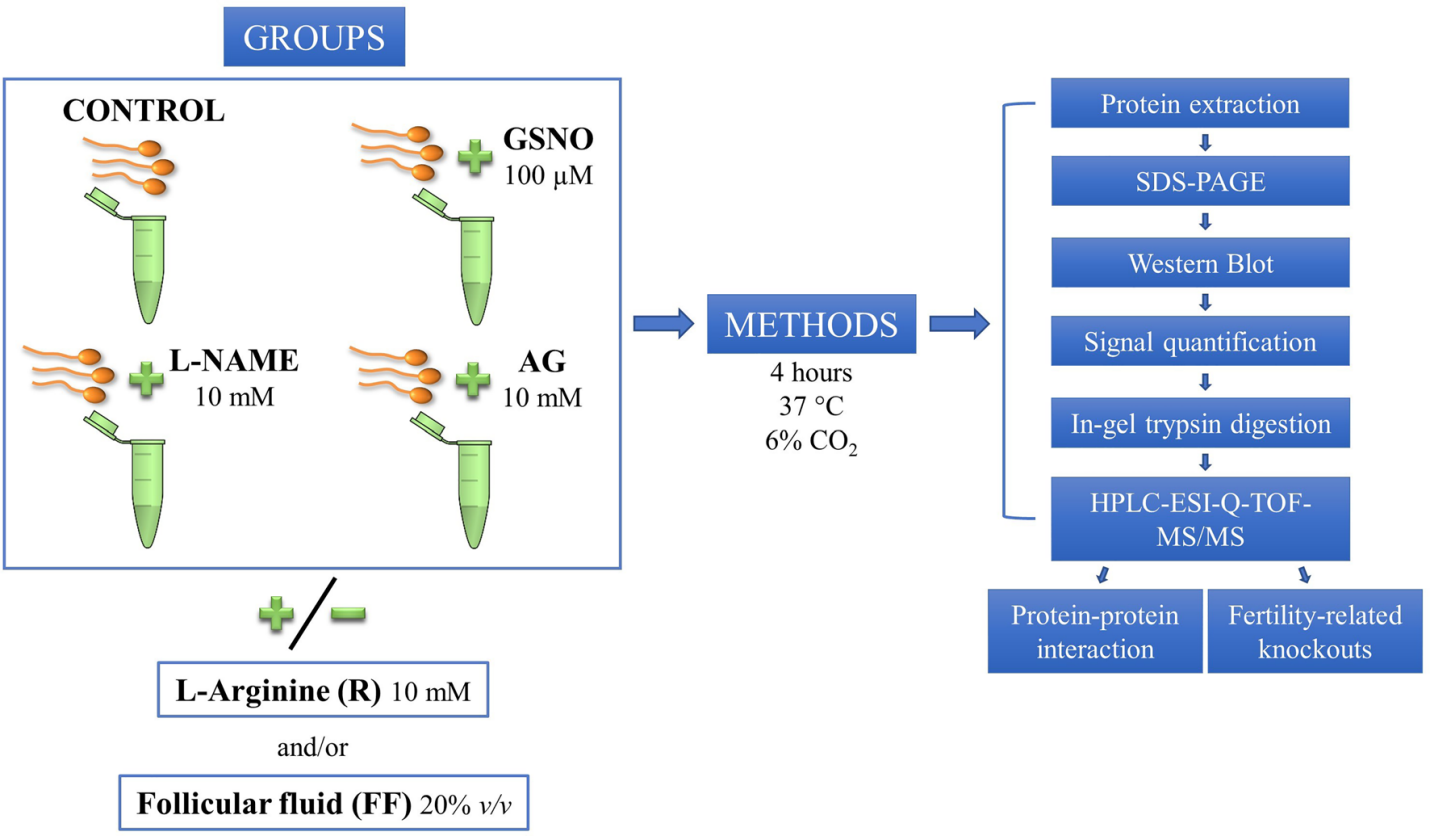
Experimental design. Human spermatozoa were capacitated for 4 h in the presence/absence of a NO donor (GSNO) and two NOS inhibitors (l-NAME and AG). The experimental groups were supplemented or not with L-Arginine and/or follicular fluid (FF). Sperm proteins were subjected to electrophoresis followed by Western Blot to analyze the phosphorylation levels of phospho-PKA substrates (PKAs-P) and tyrosine residues (Tyr-P). The amount of signal in each membrane was determined by chemiluminescence and, subsequently, quantified. Specific protein bands, that showed significant differences amongst the treatments mentioned above, were subjected to in-gel trypsin digestion, followed by mass spectrometry analysis (HPLC-ESI-Q-TOF–MS/MS). Testis-specific protein–protein interaction and fertility-related knockout phenotypes were also analyzed.

To investigate how NO modulates the phosphorylation of PKA substrates and tyrosine residues, sperm samples were capacitated in Sydney IVF Sperm Medium for 4 h at 37 °C and 6% CO_2_ with different treatments (Fig. 7). The following experimental groups were used: CONTROL: spermatozoa incubated in the absence of any treatment; GSNO: spermatozoa incubated with 100 µM S-Nitrosoglutathione, a NO donor; l-NAME: spermatozoa incubated with 10 mM N^G^-Nitro-l-Arginine Methyl Ester Hydrochloride, a NOS inhibitor; AG: spermatozoa incubated with 10 mM Aminoguanidine Hemisulfate salt, another NOS inhibitor.

To determine whether the addition of l-Arginine, the substrate for NO synthesis, and FF has an effect on phospho-PKA substrates and Tyr-P, the capacitation medium was supplemented or not with 10 mM l-Arginine and/or 20% *v*/*v* FF (Fig. 7). The concentrations of the different treatments were selected based on the scientific literature^12,74–77^.

The experimental groups mentioned above were subjected to WB analysis to detect differences in the levels of phospho-PKA substrates and Tyr-P. The protein bands that showed significant differences amongst the treatments were isolated, digested with trypsin and analyzed via mass spectrometry, to identify their proteomic profile.

Data regarding testis-specific PPI and fertility-related knockout phenotypes were analyzed to predict the putative consequences derived from a lack of biological activity as result of modification in the protein phosphorylation degree.

### Materials

Unless otherwise stated, chemicals and reagents were purchased from Sigma-Aldrich Quimica S.A. (Madrid, Spain). Sydney IVF Sperm Medium was provided by Cook Medical (Barcelona, Spain). 4–15% polyacrylamide gels were supplied by Bio-Rad (Madrid, Spain) and PVDF membranes by Merck (Madrid, Spain). The primary antibodies were: anti-phospho-PKA substrates (PKAs-P) (Cell Signaling Technology, Beverly, USA, #9624), anti-phosphotyrosine (Tyr-P) (Abcam, Cambridge, UK, #ab10321) and anti-β-tubulin (β-TUB) (Sigma-Aldrich Quimica S.A., Madrid, Spain, #T0198)^78,79^. Horseradish peroxidase-conjugated anti-rabbit and anti-mouse IgGs were obtained from Santa Cruz Biotechnology (Heidelberg, Germany, #sc2004) and Bio-Rad (Madrid, Spain, #1706516), respectively. PageBlue was provided by Thermo Scientific (Rockford, IL, USA), whereas acetonitrile, trifluoroacetic acid and formic acid by Fisher Scientific (UK). Trypsin Gold Proteomics Grade and ProteaseMax surfactant were purchased from Promega Corporation (Madison, MI, USA).

### Sperm samples

Semen samples were obtained by masturbation after 3–5 days of sexual abstinence from 7 donors. All samples were allowed to liquefy for at least 30 min at 37 °C; then they were evaluated for sperm concentration, motility, and morphology according to the World Health Organization guidelines (2010)^21,80^. Only specimens with normal parameters were used in the experiments^21,80^.

### FF samples

FF samples were obtained from 26 women taking part in the oocyte donation program at IVI-RMA Global (Murcia, Spain). Ovarian stimulation was achieved by administering a human recombinant follicle-stimulating hormone and a gonadotrophin-releasing hormone antagonist, as previously described^81^. When the follicles reached an average diameter of 17.5–18 mm, the ovulation was triggered with a GnRH agonist^81^. Approximately thirty-six hours later, dominant follicles were punctured transvaginally under ultrasound guidance, and FF was aspirated together with the oocyte. After oocyte retrieval, only visibly blood-free FF samples (*n* = 26) were further processed^82^. FF was centrifuged during 15 min at 1500×*g*. The supernatant was filtered using 0.22 µm filter units (Merck KGaA, Darmstadt, Germany) to remove cellular debris, then aliquoted and stored at -20 °C until use. Before and after centrifugation, an aliquot from each FF sample was used to determine hemoglobin (Hb) levels with a HemoCue Plasma/Low Hb System (Ängelholm, Sweden) to ensure that, after being processed, the FF did not contain detectable levels of Hb (Supplementary Table S6), which is a NO scavenger^83^.

### SDS-PAGE and Western Blot (WB)

Sperm protein extracts were obtained, separated by electrophoresis and immunoblotted as previously described^78,84^. Briefly, spermatozoa were collected by centrifugation, washed in 600 µL of phosphate buffer solution without calcium chloride and magnesium chloride (PBS), resuspended in Laemmli sample buffer^85^, boiled for 5 min and centrifuged once more. Supernatants were then supplemented with 5% *v/v* β-mercaptoethanol and boiled again for 3 min. Next, the protein extracts equivalent to 1 × 10^6^ sperm were loaded per lane, subjected to SDS-PAGE at 80 mA and electro-transferred to PVDF membranes at 250 mA for 75 min on ice. For PKAs-P and β-TUB immunodetections, membranes were blocked for 1 h at room temperature with 5% *w/v* bovine serum albumin (BSA) in TBS containing 0.1% *v/v* Tween 20 (TTBS), whereas for Tyr-P a 5% *w/v* bovine serum albumin (BSA) in PBS containing 0.1% *v/v* Tween 20 (TPBS) was used for the same time. Incubations with the primary antibodies were performed as follows: 1:2,000 in 5% *w/v* BSA-TTBS (overnight at 4 °C) for PKAs-P; 1:10,000 in 1% *w/v* BSA-TPBS (2 h at room temperature) for Tyr-P; 1:5,000 in 1% *w/v* BSA-TTBS for β-TUB (overnight at 4 °C). As far as the secondary antibodies are concerned, the incubations were done as follows: anti-rabbit, 1:10,000 in 5% *w/v* BSA-TTBS (2 h at room temperature) for PKAs-P; anti-mouse, 1:10,000 in 1% *w/v* BSA-TPBS (1 h at room temperature) for Tyr-P; 1:10,000 in 1% *w/v* BSA-TTBS (1 h at room temperature) for β-TUB. After each antibody incubation, the membranes were washed 3 × 5 min with either TTBS or TPBS. When necessary, PVDF membranes were stripped at 60 °C for 20 min in 2% *w/v* SDS, 0.74% *v/v* β-mercaptoethanol, 62.5 mM Tris, pH 6.5, and washed 5 × 5 min in TTBS. Specifically, the stripping procedure was first performed after the PKAs-P immunodetection, followed by a second stripping step after the Tyr-P immunodetection. Blots were visualized by chemiluminescence (Amersham Imager 600, GE Healthcare Life Sciences, Buckinghamshire, UK) using a Pierce® ECL 2 Western Blotting Substrate (80,196, Lumigen Inc, Southfield, MI, USA) according to the manufacturer's instructions. The relative amount of signal in each membrane was quantified using the ImageQuant TL v8.1 software (GE Healthcare). β-TUB was used as a loading control and the signal for each lane was normalized to its corresponding β-TUB value, as follows: firstly, the arbitrary value of 1 (or 100% intensity) was assigned to the β-TUB relative optical density (ROD) value of the CONTROL group; secondly, the corresponding β-TUB ROD value of all other experimental groups was calculated in relation to the CONTROL value of 1 (or 100% intensity) to determine how much higher, or lower is the β-TUB ROD value in the experimental group vs CONTROL; thirdly, the signal for each WB lane was normalized by dividing the lane’s ROD by its corresponding β-TUB value, previously calculated in the second step. Molecular masses were expressed in kilodaltons (kD).

### SDS-PAGE gel staining and in-gel trypsin digestion

Once the immunoblots were analyzed, a sample of sperm protein extracts was subjected to electrophoresis, as described in the previous section, followed by gel staining. Briefly, the gel was washed 3 × 10 min with Milli-Q water and stained overnight at room temperature with PageBlue. Next, to destain the gel, it was rinsed twice and then washed for 4 × 15 min with Milli-Q water.

The selected protein bands were spliced in approximately 2 × 2 mm parts and prepared for the digestion process as previously reported^86,87^. The bands were washed twice with Milli-Q water and then twice with 25 mM ammonium bicarbonate buffer pH 8.5 in 50% *v/v* acetonitrile during 30 min at 37 °C. After removing the supernatant, bands were dried for 15 min using an Eppendorf 5301 vacuum evaporator, and then they were incubated with 100 µL of 25 mM ammonium bicarbonate buffer pH 8.5 with 20 mM DTT at 56 °C for 20 min. The supernatant was removed and the samples were alkylated by adding 100 µL of 25 mM ammonium bicarbonate buffer pH 8.5 with 100 mM iodoacetamide during 30 min at room temperature in the dark. The supernatant was again removed and the gel spots were washed first with 25 mM ammonium bicarbonate buffer pH 8.5 and then with 25 mM ammonium bicarbonate buffer pH 8.5 in 50% *v/v* acetonitrile during 15 min at 37 °C each time. After washing, the gel spots were dried again and then incubated with 50 µL 25 mM ammonium bicarbonate buffer pH 8.5 containing 0.5 µg of Trypsin Gold Proteomics Grade and 0.01% *w/v* ProteaseMax surfactant during 10 min at 4 °C. Next, the samples were submitted to digestion during at least 3 h at 37 °C. The supernatant was collected in a new tube and evaporated. To enhance the extraction of digested fragments from the remaining gel spots, they were washed with 100 µL of a solution containing 50% *v/v* acetonitrile and 0.5% *v/v* trifluoroacetic acid and then with 100 µL of acetonitrile during 30 min at 37 °C each time. Finally, after these washing steps, both supernatants were collected in the same tube and dried using the vacuum evaporator.

### HPLC-ESI-Q-TOF–MS/MS analysis

The separation and analysis of the tryptic digests of the samples were performed as previously described^86,87^, with a HPLC/MS system consisting of an Agilent 1290 Infinity II Series HPLC (Agilent Technologies, Santa Clara, CA, USA) equipped with an Automated Multisampler Module and a High-Speed Binary Pump, connected to an Agilent 6550 Q-TOF Mass Spectrometer (Agilent Technologies, Santa Clara, CA, USA) using an Agilent Jet Stream Dual electrospray (AJS-Dual ESI) interface. Experimental parameters for HPLC and Q-TOF were set in the MassHunter Workstation Data Acquisition software (Agilent Technologies, Rev. B.08.00).

Dry samples from trypsin digestion were resuspended in 20 µL of buffer A, consisting in water/acetonitrile/formic acid (94.9:5:0.1). The samples were injected onto an Agilent AdvanceBio Peptide Mapping HPLC column (2.7 µm, 100 × 2.1 mm, Agilent Technologies), thermostated at 55 °C, at a flow rate of 0.4 mL/min. After the injection, the column was washed with buffer A for 2 min and the digested peptides were eluted using a linear gradient 0–40% with buffer B (water/acetonitrile/formic acid, 10:89.9:0.1) for 30 min.

The mass spectrometer was operated in the positive mode. The nebulizer gas pressure was set to 35 psi, whereas the drying gas flow was set to 14 L/min at a temperature of 300 °C, and the sheath gas flow was set to 11 L/min at a temperature of 250 ºC. The capillary spray, fragmentor and octopole RF Vpp voltages were 3500 V, 360 V and 750 V, respectively. Profile data were acquired for both MS and MS/MS scans in extended dynamic range mode. MS and MS/MS mass range were 50–1700 m/z and scan rates were 8 spectra/sec for MS and 3 spectra/sec for MS/MS. Auto MS/MS mode was used with precursor selection by abundance and a maximum of 20 precursors were selected per cycle. A ramped collision energy was used with a slope of 3.6 and an offset of -4.8. The same ion was rejected after two consecutive scans.

Data processing and analysis was performed with the Spectrum Mill MS Proteomics Workbench software (Rev B.06.00.201, Agilent Technologies, Santa Clara, CA, USA). Briefly, raw data were extracted under default conditions as follows: unmodified or carbamidomethylated cysteines; [MH] + 50–10,000 m/z; maximum precursor charge + 5; minimum signal-to-noise MS (S/N) 25; finding ^12^C signals.

The MS/MS search against the appropriate and updated protein database was performed with the following criteria: variable modifications search mode (carbamidomethylated cysteines, STY phosphorylation, oxidized methionine, and N-terminal glutamine conversion to pyroglutamic acid); tryptic digestion with 5 maximum missed cleavages; ESI-Q-TOF instrument; minimum matched peak intensity 50%; maximum ambiguous precursor charge + 5; monoisotopic masses; peptide precursor mass tolerance 20 ppm; product ion mass tolerance 50 ppm; and calculation of reversed database scores. Validation of peptide and protein data was performed using auto thresholds. General confidence criteria for peptide validation were: score > 9; SPI > 70%.

### Protein–protein interactome network and knockout phenotypes related to sperm proteins affected by phosphorylation

To reinforce the biological relevance of the results, protein–protein interactions (PPI) were analyzed using NetworkAnalyst 3.0, with the purpose to identify key factors acting as hubs, based on connectivity of the nodes (degree, i.e. number of connections of the nodes). Only testis-specific PPI, using data available through the DifferentialNet database (http://netbio.bgu.ac.il/diffnet/) were used.

The genes that encode the proteins identified in the selected WB bands were inserted in the Mouse Genome Informatics database (http://www.informatics.jax.org/). A search was performed, using the MGI Batch Query tool, for fertility-related knockout phenotypes in mammals, available to the current date.

### Statistical analysis

Relative amounts of signal in each WB are presented as the mean ± standard error of the mean (SEM) for each experimental group. The Kolmogorov–Smirnov and Levene tests were used to check our data for normality and homogeneity of variance, respectively. One-Way ANOVA followed by Tukey test were used to analyze differences between mean values of multiple groups. The level of significance was set at *P* < 0.05. All statistical analyses were conducted using IBM SPSS Statistics for Windows, Version 20.0 (IBM, Armonk, NY, USA).

### Ethics approval

This study was approved by the Ethics Review Committee of CEIC Hospital General Universitario Jose Maria Morales Meseguer (Murcia, Spain) (Approval No. EST: 06/17) and registered at https://clinicaltrials.gov/ (ID: NCT03307655). Men and women were recruited from the gamete donation program at IVI-RMA Global (Murcia, Spain), after providing their signed informed consent. All experiments were performed in accordance with relevant guidelines and regulations.

## Supplementary Information

Supplementary Information.

## Data Availability

All data generated or analyzed during this study are included in this published article (and its Supplementary Information file).

## References

[1] Pacher P, Beckman JS, Liaudet L (2007). Nitric oxide and peroxynitrite in health and disease. Physiol. Rev..

[2] Rosselli M, Keller PJ, Dubey RK (1998). Role of nitric oxide in the biology, physiology and pathophysiology of reproduction. Hum. Reprod. Update.

[3] Staicu F-D, Matas Parra C, Saravi SSS (2017). Nitric oxide: Key features in spermatozoa. Nitric Oxide Synthase—Simple Enzyme-Complex Roles.

[4] O’Bryan MK, Zini A, Cheng CY, Schlegel PN (1998). Human sperm endothelial nitric oxide synthase expression: Correlation with sperm motility. Fertil. Steril..

[5] Herrero MB, Pérez Martínez S, Viggiano JM, Polak JM, de Gimeno MF (1996). Localization by indirect immunofluorescence of nitric oxide synthase in mouse and human spermatozoa. Reprod. Fertil. Dev..

[6] Wang J, He Q, Yan X, Cai Y, Chen J (2014). Effect of exogenous nitric oxide on sperm motility in vitro. Biol. Res..

[7] Zini A, De Lamirande E, Gagnon C (1995). Low levels of nitric oxide promote human sperm capacitation in vitro. J. Androl..

[8] Herrero MB, Gagnon C (2001). Nitric oxide: A novel mediator of sperm function. J. Androl..

[9] Revelli A (1999). Follicular fluid proteins stimulate nitric oxide (NO) synthesis in human sperm: A possible role for NO in acrosomal reaction. J. Cell. Physiol..

[10] Donnelly ET, Lewis SE, Thompson W, Chakravarthy U (1997). Sperm nitric oxide and motility: The effects of nitric oxide synthase stimulation and inhibition. Mol. Hum. Reprod..

[11] Jin S-K, Yang W-X (2017). Factors and pathways involved in capacitation: How are they regulated?. Oncotarget.

[12] Lefièvre L (2007). Human spermatozoa contain multiple targets for protein S-nitrosylation: An alternative mechanism of the modulation of sperm function by nitric oxide?. Proteomics.

[13] Belén Herrero M, Chatterjee S, Lefièvre L, De Lamirande E, Gagnon C (2000). Nitric oxide interacts with the cAMP pathway to modulate capacitation of human spermatozoa. Free Radic. Biol. Med..

[14] Herrero MB, de Lamirande E, Gagnon C (1999). Nitric oxide regulates human sperm capacitation and protein-tyrosine phosphorylation in vitro. Biol. Reprod..

[15] Visconti PE (2002). Novel signaling pathways involved in sperm acquisition of fertilizing capacity. J. Reprod. Immunol..

[16] McVey M, Hill J, Howlett A, Klein C (1999). Adenylyl cyclase, a coincidence detector for nitric oxide. J. Biol. Chem..

[17] Murad F (1994). The nitric oxide-cyclic GMP signal transduction system for intracellular and intercellular communication. Recent Prog. Horm. Res..

[18] Lohmann SM, Vaandrager AB, Smolenski A, Walter U, De Jonge HR (1997). Distinct and specific functions of cGMP-dependent protein kinases. Trends Biochem. Sci..

[19] Pfeifer A (1999). Structure and function of cGMP-dependent protein kinases. Rev. Physiol. Biochem. Pharmacol..

[20] Revelli A (2001). Signaling pathway of nitric oxide-induced acrosome reaction in human spermatozoa. Biol. Reprod..

[21] Miraglia E (2011). Nitric oxide stimulates human sperm motility via activation of the cyclic GMP/protein kinase G signaling pathway. Reproduction.

[22] Bender AT, Beavo JA (2006). Cyclic nucleotide phosphodiesterases: Molecular regulation to clinical use. Pharmacol. Rev..

[23] Conti M, Beavo J (2007). Biochemistry and physiology of cyclic nucleotide phosphodiesterases: Essential components in cyclic nucleotide signaling. Annu. Rev. Biochem..

[24] Beavo JA (1995). Cyclic nucleotide phosphodiesterases: Functional implications of multiple isoforms. Physiol. Rev..

[25] Barroso G (1999). Vascular endothelial growth factor, nitric oxide, and leptin follicular fluid levels correlate negatively with embryo quality in IVF patients. Fertil. Steril..

[26] O’Flaherty C, de Lamirande E, Gagnon C (2006). Positive role of reactive oxygen species in mammalian sperm capacitation: Triggering and modulation of phosphorylation events. Free Radic. Biol. Med..

[27] Xia J, Benner MJ, Hancock REW (2014). NetworkAnalyst–integrative approaches for protein–protein interaction network analysis and visual exploration. Nucleic Acids Res..

[28] Stival C (2016). Sperm capacitation and acrosome reaction in mammalian sperm. Adv. Anat. Embryol. Cell Biol..

[29] Tosti E, Ménézo Y (2016). Gamete activation: basic knowledge and clinical applications. Hum. Reprod. Update.

[30] Thundathil J, de Lamirande E, Gagnon C (2003). Nitric oxide regulates the phosphorylation of the threonine–glutamine–tyrosine motif in proteins of human spermatozoa during capacitation. Biol. Reprod..

[31] Rahman MS (2014). Sodium nitroprusside suppresses male fertility in vitro. Andrology.

[32] Ardito F, Giuliani M, Perrone D, Troiano G, Muzio LL (2017). The crucial role of protein phosphorylation in cell signaling and its use as targeted therapy (Review). Int. J. Mol. Med..

[33] Aitken RJ, Paterson M, Fisher H, Buckingham DW, van Duin M (1995). Redox regulation of tyrosine phosphorylation in human spermatozoa and its role in the control of human sperm function. J. Cell Sci..

[34] Leclerc P, de Lamirande E, Gagnon C (1997). Regulation of protein-tyrosine phosphorylation and human sperm capacitation by reactive oxygen derivatives. Free Radic. Biol. Med..

[35] Leclerc P, de Lamirande E, Gagnon C (1998). Interaction between Ca2+, cyclic 3′,5′ adenosine monophosphate, the superoxide anion, and tyrosine phosphorylation pathways in the regulation of human sperm capacitation. J. Androl..

[36] Roy SC, Atreja SK (2008). Tyrosine phosphorylation of a 38-kDa capacitation-associated buffalo (*Bubalus bubalis*) sperm protein is induced by l-arginine and regulated through a cAMP/PKA-independent pathway. Int. J. Androl..

[37] O’Flaherty C, Rodriguez P, Srivastava S (2004). l-Arginine promotes capacitation and acrosome reaction in cryopreserved bovine spermatozoa. Biochim. Biophys. Acta.

[38] de Andrade AFC (2018). Nitric oxide in frozen-thawed equine sperm: Effects on motility, membrane integrity and sperm capacitation. Anim. Reprod. Sci..

[39] Pfeiffer S, Leopold E, Schmidt K, Brunner F, Mayer B (1996). Inhibition of nitric oxide synthesis by NG-nitro-l-arginine methyl ester (l-NAME): Requirement for bioactivation to the free acid NG-nitro-l-arginine. Br. J. Pharmacol..

[40] Boer R (2000). The inhibitory potency and selectivity of arginine substrate site nitric-oxide synthase inhibitors is solely determined by their affinity toward the different isoenzymes. Mol. Pharmacol..

[41] Misko TP (1993). Selective inhibition of the inducible nitric oxide synthase by aminoguanidine. Eur. J. Pharmacol..

[42] Munuce MJ, Caille AM, Botti G, Berta CL (2004). Modulation of human sperm function by follicular fluid. Andrologia.

[43] Benoff S (1997). Carbohydrates and fertilization: An overview. Mol. Hum. Reprod..

[44] Yao YQ, Chiu CN, Ip SM, Ho PC, Yeung WS (1998). Glycoproteins present in human follicular fluid that inhibit the zona- binding capacity of spermatozoa. Hum. Reprod..

[45] Chiu PCN (2003). Zona-binding inhibitory factor-1 from human follicular fluid is an isoform of glycodelin. Biol. Reprod..

[46] Chiu PCN (2004). The contribution of d-mannose, l-fucose, N-acetylglucosamine, and selectin residues on the binding of glycodelin isoforms to human spermatozoa. Biol. Reprod..

[47] Redgrove KA (2011). Involvement of multimeric protein complexes in mediating the capacitation-dependent binding of human spermatozoa to homologous zonae pellucidae. Dev. Biol..

[48] Dun MD (2011). The chaperonin containing TCP1 complex (CCT/TRiC) is involved in mediating sperm-oocyte interaction. J. Biol. Chem..

[49] Panner Selvam MK, Agarwal A, Pushparaj PN (2019). A quantitative global proteomics approach to understanding the functional pathways dysregulated in the spermatozoa of asthenozoospermic testicular cancer patients. Andrology.

[50] Vijayaraghavan S, Goueli SA, Davey MP, Carr DW (1997). Protein kinase A-anchoring inhibitor peptides arrest mammalian sperm motility. J. Biol. Chem..

[51] Miki K (2002). Targeted disruption of the Akap4 gene causes defects in sperm flagellum and motility. Dev. Biol..

[52] Reddy RK, Lu J, Lee AS (1999). The endoplasmic reticulum chaperone glycoprotein GRP94 with Ca(2+)-binding and antiapoptotic properties is a novel proteolytic target of calpain during etoposide-induced apoptosis. J. Biol. Chem..

[53] Walsh A (2008). Identification of the molecular Chaperone, heat shock protein 1 (Chaperonin 10), in the reproductive tract and in capacitating spermatozoa in the male mouse1. Biol. Reprod..

[54] Dasgupta SK, Thiagarajan P (2018). Wdr-1 is essential for F-actin interaction with focal adhesions in platelets. Blood Coagul. Fibrinolysis.

[55] Breitbart H, Finkelstein M (2018). Actin cytoskeleton and sperm function. Biochem. Biophys. Res. Commun..

[56] Breitbart H, Cohen G, Rubinstein S (2005). Role of actin cytoskeleton in mammalian sperm capacitation and the acrosome reaction. Reproduction.

[57] Wanderling S (2007). GRP94 is essential for mesoderm induction and muscle development because it regulates insulin-like growth factor secretion. Mol. Biol. Cell.

[58] Mao C (2010). Targeted mutation of the mouse grp94gene disrupts development and perturbs endoplasmic reticulum stress signaling. PLoS ONE.

[59] Kile BT (2007). Mutations in the cofilin partner Aip1/Wdr1 cause autoinflammatory disease and macrothrombocytopenia. Blood.

[60] du Plessis SS, Agarwal A, Mohanty G, van der Linde M (2015). Oxidative phosphorylation versus glycolysis: What fuel do spermatozoa use?. Asian J. Androl..

[61] Storey BT (2008). Mammalian sperm metabolism: Oxygen and sugar, friend and foe. Int. J. Dev. Biol..

[62] Ficarro S (2003). Phosphoproteome analysis of capacitated human sperm: Evidence of tyrosine phosphorylation of a kinase-anchoring protein 3 and valosin-containing protein/p97 during capacitation. J. Biol. Chem..

[63] Bartolome F (2013). Pathogenic VCP mutations induce mitochondrial uncoupling and reduced ATP levels. Neuron.

[64] Gupta V, Bamezai RNK (2010). Human pyruvate kinase M2: A multifunctional protein. Protein Sci..

[65] Qin Z (2018). Aconitate 2 (ACO2) and pyruvate kinase M2 (PKM2) are good predictors of human sperm freezability. Int. J. Clin. Exp. Med..

[66] Ji Z-L (2012). Association of heat shock proteins, heat shock factors and male infertility. Asian Pac. J. Reprod..

[67] Huszar G, Stone K, Dix D, Vigue L (2000). Putative creatine kinase M-isoform in human sperm is identifiedas the 70-kilodalton heat shock protein HspA2. Biol. Reprod..

[68] Huszar G (2007). Fertility testing and ICSI sperm selection by hyaluronic acid binding: Clinical and genetic aspects. Reprod. Biomed. Online.

[69] Han C (2011). Identification of heat shock protein 5, calnexin and integral membrane protein 2B as Adam7-interacting membrane proteins in mouse sperm. J. Cell. Physiol..

[70] Bromfield EG, Nixon B (2013). The function of chaperone proteins in the assemblage of protein complexes involved in gamete adhesion and fusion processes. Reproduction.

[71] Boilard M (2004). Localization of the chaperone proteins GRP78 and HSP60 on the luminal surface of bovine oviduct epithelial cells and their association with spermatozoa. Biol. Reprod..

[72] Töpfer-Petersen E (2002). Function of the mammalian oviductal sperm reservoir. J. Exp. Zool..

[73] Buzadzic B (2015). New insights into male (in)fertility: The importance of NO. Br. J. Pharmacol..

[74] Romero-Aguirregomezcorta J, Santa ÁP, García-Vázquez FA, Coy P, Matás C (2014). Nitric oxide synthase (NOS) inhibition during porcine in vitro maturation modifies oocyte protein S-nitrosylation and in vitro fertilization. PLoS ONE.

[75] Leal ACMS, Caldas-Bussiere MC, de Carvalho CSP, Viana KS, Quirino CR (2009). Role of nitric oxide on quality of freshly ejaculated bull spermatozoa during heparin-induced in vitro capacitation. Anim. Reprod. Sci..

[76] Calvo L (1989). Follicular fluid-induced acrosome reaction distinguishes a subgroup of men with unexplained infertility not identified by semen analysis. Fertil. Steril..

[77] Getpook C, Wirotkarun S (2007). Sperm motility stimulation and preservation with various concentrations of follicular fluid. J. Assist. Reprod. Genet..

[78] Soriano-Úbeda C, García-Vázquez FA, Romero-Aguirregomezcorta J, Matás C (2017). Improving porcine in vitro fertilization output by simulating the oviductal environment. Sci. Rep..

[79] Řimnáčová H (2020). Low doses of Bisphenol S affect post-translational modifications of sperm proteins in male mice. Reprod. Biol. Endocrinol..

[80] Patel A, Leong J, Ramasamy R (2017). Prediction of male infertility by the World Health Organization laboratory manual for assessment of semen analysis: A systematic review. Arab J. Urol..

[81] Melo M (2009). GnRH agonist versus recombinant HCG in an oocyte donation programme: A randomized, prospective, controlled, assessor-blind study. Reprod. Biomed. Online.

[82] Staicu F-D (2021). Nitrite and nitrate levels in follicular fluid from human oocyte donors are related to ovarian response and embryo quality. Front. Cell Dev. Biol..

[83] Azarov I (2005). Nitric oxide scavenging by red blood cells as a function of hematocrit and oxygenation. J. Biol. Chem..

[84] Navarrete FA (2015). Biphasic role of calcium in mouse sperm capacitation signaling pathways. J. Cell. Physiol..

[85] Laemmli UK (1970). Cleavage of structural proteins during the assembly of the head of bacteriophage T4. Nature.

[86] Stetson I (2015). Four glycoproteins are expressed in the cat zona pellucida. Theriogenology.

[87] Vieira L, Matás C, Torrecillas A, Saez F, Gadea J (2021). Seminal plasma components from fertile stallions involved in the epididymal sperm freezability. Andrology.

[88] Heberle H, Meirelles GV, da Silva FR, Telles GP, Minghim R (2015). InteractiVenn: A web-based tool for the analysis of sets through Venn diagrams. BMC Bioinformatics.

