# Supplementary material for: Nitric oxide-targeted protein phosphorylation during human sperm capacitation
Source: Sci Rep. 2021 Oct 25;11:20979. doi: 10.1038/s41598-021-00494-1 (PMC8546126; doi:10.1038/s41598-021-00494-1)
Supplement: Supplementary file 1 — Supplementary Information. [file 41598_2021_494_MOESM1_ESM.pdf]

Nitric oxide-targeted protein phosphorylation during human sperm capacitation

Authors and affiliations: Florentin-Daniel Staicu [1,2], Juan Carlos Martínez-Soto [2,3], Sebastian Canovas [2,4] and Carmen Matás [1,2,*]

[1] Department of Physiology, Veterinary Faculty, University of Murcia, International Excellence Campus for Higher Education and Research (Campus Mare Nostrum), Murcia, Spain.

[2] Institute for Biomedical Research of Murcia (IMIB), Murcia, Spain.

[3] IVI-RMA Global, Murcia, Spain.

[4] Department of Physiology, Nursery Faculty, University of Murcia, International Excellence Campus for Higher Education and Research (Campus Mare Nostrum), Murcia, Spain.

Keywords: nitric oxide; human follicular fluid; human sperm; capacitation; protein phosphorylation.

[*] Corresponding author: Carmen Matás, Department of Physiology, Veterinary Faculty, University of Murcia, Calle Campus Universitario, 11, 30100 Murcia, Spain. E-mail: cmatas@um.es; Tel.: +34-868-88-7256.

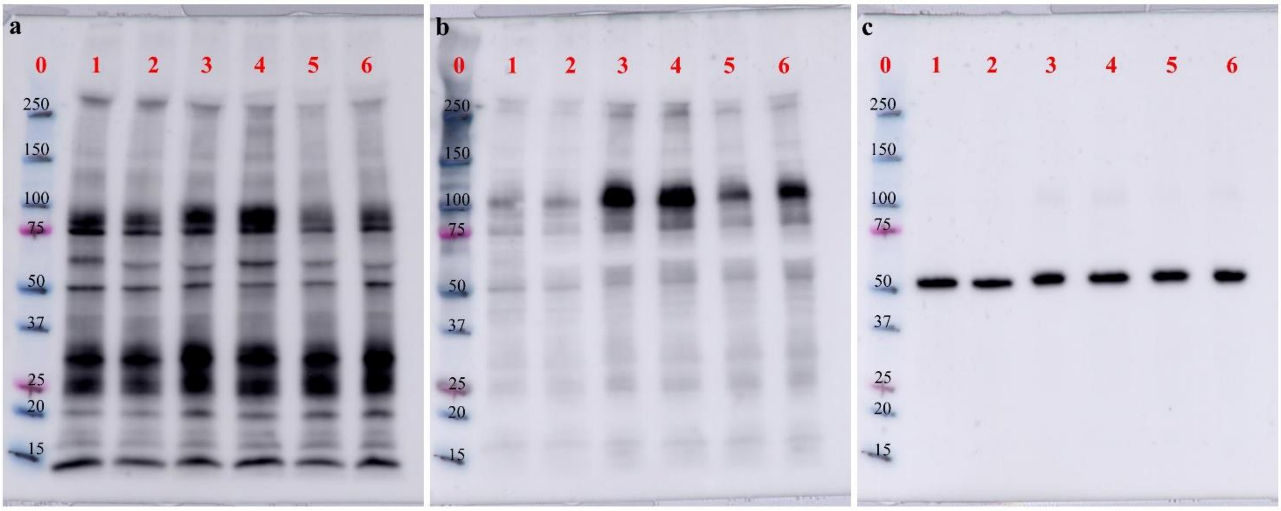

Supplementary Fig. S1. Grouping of representative membranes for phospho-PKA substrates (a), tyrosine phosphorylation (b) and β-tubulin (c), individually delineated by white space. Red numbers indicate the content of each lane. (**0**) Precision Plus Protein™ Dual Color Standards (Bio-Rad, Madrid, Spain, #1610374). Molecular weights are expressed in kilodaltons. (**1-6**) Immunoblotted protein extracts from spermatozoa incubated under different experimental conditions, as follows: (**1**) Dulbecco's Phosphate-Buffered Saline without calcium chloride and magnesium chloride, time 0 h. (**2**) Sperm medium, time 0 h. (**3**) CONTROL: sperm medium, time 4 h. (**4**) GSNO: sperm medium supplemented with 100 µM S-Nitrosoglutathione, time 4 h. (**5**) L-NAME: sperm medium supplemented with 10 mM N^G-Nitro-L-arginine Methyl Ester Hydrochloride, time 4 h. (**6**) AG: sperm medium supplemented with 10 mM Aminoguanidine Hemisulfate salt, time 4 h.

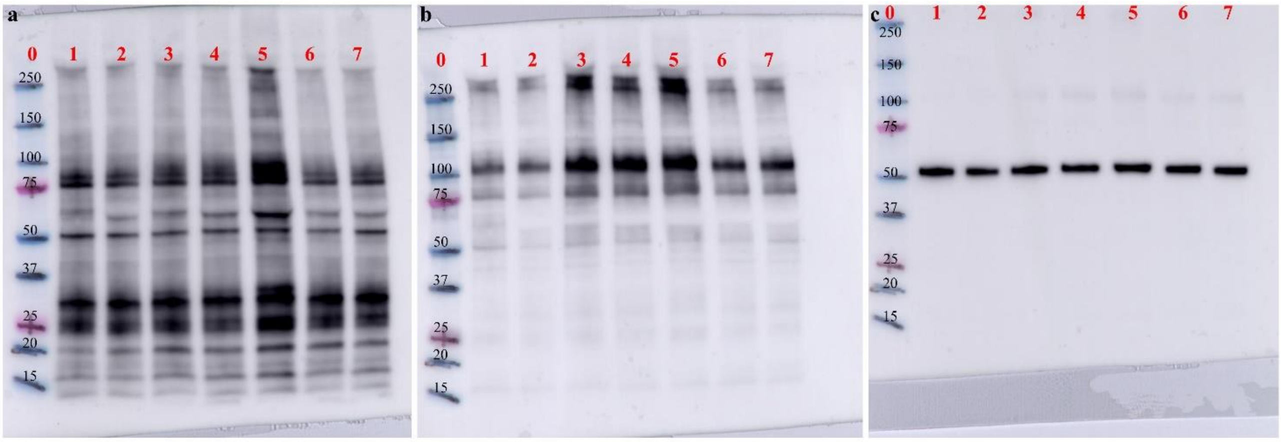

Supplementary Fig. S2. Grouping of representative membranes for phospho-PKA substrates (a), tyrosine phosphorylation (b) and β-tubulin (c), individually delineated by white space. Red numbers indicate the content of each lane. (**0**) Precision Plus Protein^{TM} Dual Color Standards (Bio-Rad, Madrid, Spain, #1610374). Molecular weights are expressed in kilodaltons. (**1-7**) Immunoblotted protein extracts from spermatozoa incubated under different experimental conditions, as follows: (**1**) Dulbecco's Phosphate-Buffered Saline without calcium chloride and magnesium chloride, time 0 h. (**2**) Sperm medium, time 0 h. (**3**) CONTROL: sperm medium, time 4 h. (**4**) SM + R: sperm medium supplemented with 10 mM L-Arginine, time 4 h. (**5**) GSNO + R: sperm medium supplemented with 100 µM S-Nitrosoglutathione and 10 mM L-Arginine, time 4 h. (**6**) L-NAME + R: sperm medium supplemented with 10 mM N^{G}-Nitro-L-arginine Methyl Ester Hydrochloride and 10 mM L-Arginine, time 4 h. (**7**) AG + R: sperm medium supplemented with 10 mM Aminoguanidine Hemisulfate salt and 10 mM L-Arginine, time 4 h.

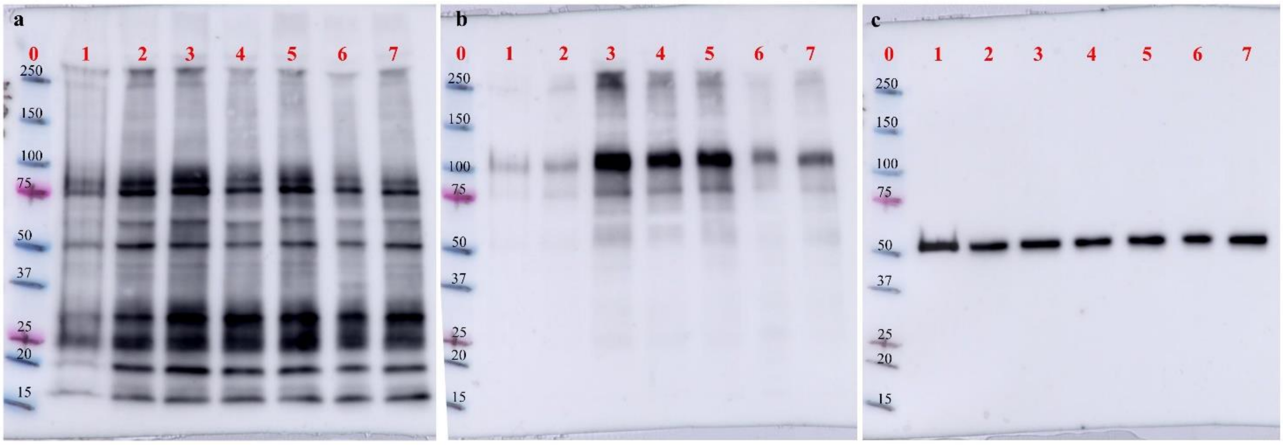

Supplementary Fig. S3. Grouping of representative membranes for phospho-PKA substrates (a), tyrosine phosphorylation (b) and β-tubulin (c), individually delineated by white space. Red numbers indicate the content of each lane. (**0**) Precision Plus Protein™ Dual Color Standards (Bio-Rad, Madrid, Spain, #1610374). Molecular weights are expressed in kilodaltons. (**1-7**) Immunoblotted protein extracts from spermatozoa incubated under different experimental conditions, as follows: (**1**) Dulbecco's Phosphate-Buffered Saline without calcium chloride and magnesium chloride, time 0 h. (**2**) Sperm medium, time 0 h. (**3**) CONTROL: sperm medium, time 4 h. (**4**) SM + FF: sperm medium supplemented with 20% *v/v* follicular fluid, time 4 h. (**5**) GSNO + FF: sperm medium supplemented with 100 µM S-Nitrosoglutathione and 20% *v/v* follicular fluid, time 4 h. (**6**) L-NAME + FF: sperm medium supplemented with 10 mM N^G-Nitro-L-arginine Methyl Ester Hydrochloride and 20% *v/v* follicular fluid, time 4 h. (**7**) AG + FF: sperm medium supplemented with 10 mM Aminoguanidine Hemisulfate salt and 20% *v/v* follicular fluid, time 4 h.

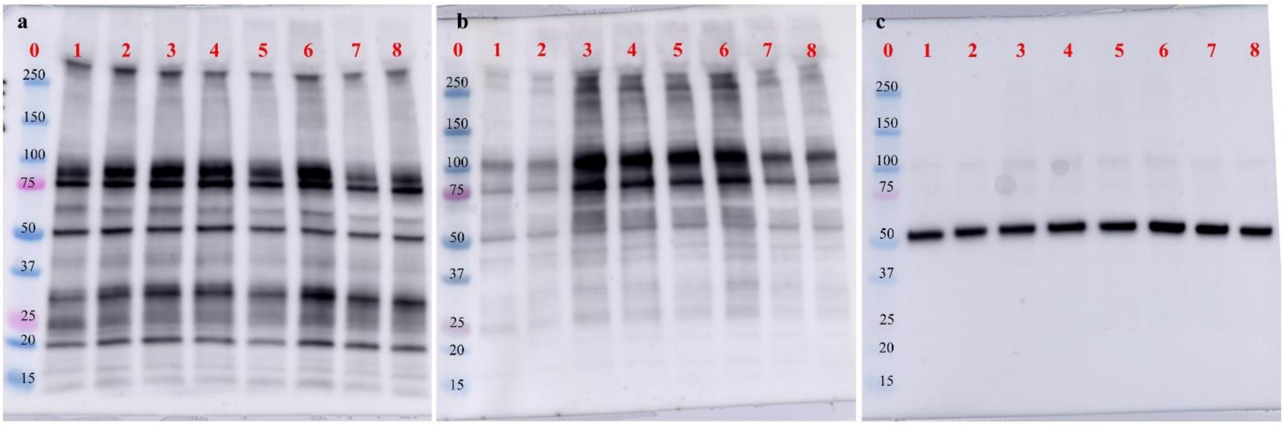

Supplementary Fig. S4. Grouping of representative membranes for phospho-PKA substrates (a), tyrosine phosphorylation (b) and β-tubulin (c), individually delineated by white space. Red numbers indicate the content of each lane. (**0**) Precision Plus Protein™ Dual Color Standards (Bio-Rad, Madrid, Spain, #1610374). Molecular weights are expressed in kilodaltons. (**1-8**) Immunoblotted protein extracts from spermatozoa incubated under different experimental conditions, as follows: (**1**) Dulbecco's Phosphate-Buffered Saline without calcium chloride and magnesium chloride, time 0 h. (**2**) Sperm medium, time 0 h. (**3**) CONTROL: sperm medium, time 4 h. (**4**) SM + R: sperm medium supplemented with 10 mM L-Arginine, time 4 h. (**5**) SM + R + FF: sperm medium supplemented with 10 mM L-Arginine and 20% *v/v* follicular fluid, time 4 h. (**6**) GSNO + R + FF: sperm medium supplemented with 100 µM S-Nitrosoglutathione, 10 mM L-Arginine and 20% *v/v* follicular fluid, time 4 h. (**7**) L-NAME + R + FF: sperm medium supplemented with 10 mM N^G-Nitro-L-arginine Methyl Ester Hydrochloride, 10 mM L-Arginine and 20% *v/v* follicular fluid, time 4 h. (**8**) AG + R + FF: sperm medium supplemented with 10 mM Aminoguanidine Hemisulfate salt, 10 mM L-Arginine and 20% *v/v* follicular fluid, time 4 h.

Supplementary Table S5. Connectivity values of the protein-protein interactome.

Factor	Degree	Betweenness centrality
HSP90AB1	287	17568443
VCP	203	13081407
HSPA5	103	4235244
PSMD2	86	4823084
ACTN1	73	5365803
HSPD1	66	2880791
CCT3	65	2572089
TCP1	58	1373968
HSPA2	54	255753
PKM2	45	1330939
CCT7	41	729093
HSP90B1	35	1147387
CCT8	34	487189
CCT6A	33	686652
PFKP	23	77117
GANAB	19	743313
CUL3	14	170261
LTF	12	757308
OBSL1	11	1118977
HDAC6	11	98247
HK1	10	313025
GABARAPL1	9	596367
FBXO25	9	592365
FN1	8	575749
TUBG1	8	376505
CDK2	8	298807
MAPK13	8	223119
CFTR	7	827248
ISG15	7	542311
CUL1	7	520754
SIRT7	7	485239
AKAP3	7	4190
ODF2	7	358831
SMURF1	7	260055
FUS	7	143663
PPP2R2B	7	90858
CDC20	7	90858
LRRK2	6	444896
GRB2	6	38842
ITGA4	6	371676
EGFR	6	36824
APP	6	258761
ESR2	6	256481
CCDC8	6	85352
SNW1	6	85352
CDC5L	6	85352

Factor	Degree	Betweenness centrality
PACRG	6	74416
MYC	6	62148
FAM86A	6	27487
CCT2	5	547947
TP63	5	522799
ESR1	5	349522
ARRB2	5	337412
AKAP4	5	2517
HSP90AA1	5	212381
GFPT1	5	153061
GABARAPL2	5	137622
MAP3K1	5	133999
FBXO6	5	29327
WDR1	5	11807
STRN4	5	8802
PPP2R4	5	8802
UBL4A	4	361798
HSPB1	4	312157
AURKA	4	188379
ATF2	4	171433
ADRB2	4	144712
MEPCE	4	140312
MDM2	4	108897
IQCB1	4	9426
AMFR	4	90618
YWHAZ	4	85298
CDK9	4	64607
MOV10	4	43681
METTL23	4	37669
PARK2	4	35135
TNFRSF1B	4	29494
RFWD2	4	2918
TBK1	4	12767
PAN2	4	12767
UBASH3B	4	12767
LGALS3BP	4	12767
CTTNBP2	4	6186
PPP4C	4	3383
NEDD1	4	3383
PRKAR2A	3	8310
HNRNPA1	3	3099
CD4	3	262434
NFKBIB	3	251812
CDK4	3	204248
SRC	3	136554
CEP76	3	117826
CEP250	3	117826
STUB1	3	109273

Factor	Degree	Betweenness centrality
UBXN6	3	96707
MIR4746	3	96707
SHC1	3	71769
CUL2	3	64534
HDAC5	3	61306
ARAF	3	58653
ANXA7	3	45758
MAPK8	3	40526
EZH2	3	34448
TYK2	3	32615
CLU	3	28651
HUWE1	3	17086
RAD21	3	16777
COPS5	3	14402
FANCA	3	9468
DOCK5	3	535
MAPK15	2	386665
CEBPA	2	282898
SVIL	2	249329
YWHAB	2	167823
RPS4X	2	167823
RPS3A	2	167823
SNORD73A	2	167823
TOMM34	2	167823
CHEK1	2	167823
STK4	2	164045
RGS6	2	132673
STIP1	2	132673
SIK2	2	87982
MCM7	2	87466
PSMD4	2	83702
BAG2	2	83702
GET4	2	83702
SHFM1	2	83702
SIRT6	2	68503
HSPE1	2	56519
BRCA1	2	56519
BAG3	2	54094
MAP1LC3A	2	54094
UNC45B	2	54094
HIPK4	2	51174
ERBB3	2	49574
METTL22	2	49574
RIPK3	2	49574
MAP3K8	2	49574
DCUN1D1	2	49574
BRAF	2	49574
ALK	2	38093

Factor	Degree	Betweenness centrality
VCAM1	2	38093
ASB4	2	38093
MAP1LC3B	2	38093
ACTB	2	38093
RAF1	2	38093
LYN	2	37454
TFE3	2	36103
SGK1	2	36103
NFKBIE	2	34415
NFKBIA	2	34415
FCHSD2	2	34415
DNAJC10	2	34415
RUVBL2	2	34415
CDH1	2	3273
CUL7	2	30824
OS9	2	25128
CD81	2	24134
NDRG1	2	18735
CCT5	2	18242
CDK5	2	18147
HLA-DRA	2	1769
TRAF3IP1	2	1769
HLA-DRB5	2	1769
HIF1A	2	1769
LDHA	2	1769
TINF2	2	1769
METTL21B	2	17086
NOS2	2	17086
PSMA3	2	17086
CEP70	2	15375
PPP1CC	2	14157
CEP57	2	14157
MAP3K3	2	10975
H2AFX	2	10551
ARRB1	2	7482
HSPA8	2	7074
SNORD14C	2	7074
SNORD14D	2	7074
FAF2	2	7007
TRAF6	2	7007
CSNK2B	2	63
CAMKMT	2	5034
SIRT3	2	4992
LDLR	2	4992
NKX3-1	2	4584
RCC1	2	4249
ELAVL1	2	3361
CBL	2	2406

Factor	Degree	Betweenness centrality
GBAS	2	2207
VDAC1	2	2207
WDR76	2	535
SNORA29	2	535
SSSCA1	2	535
ATG16L1	2	535
FAM86B1	2	334
NOTCH1	2	334
IGBP1	2	334
BTK	2	0
TUBA1A	2	0
SH3BP4	2	0
SUZ12	2	0
COIL	2	0
CAMK2A	2	0
CTNNA1	2	0
ITGB1	2	0
JAK3	2	0
RNF2	2	0
PALLD	1	0
TERF2	1	0
PSEN1	1	0
TERF1	1	0
OAS1	1	0
PHB	1	0
MICALL2	1	0
PLEK	1	0
COL17A1	1	0
DDN	1	0
MYOT	1	0
BMI1	1	0
MYOZ2	1	0
APC	1	0
CDK5R2	1	0
GIPC1	1	0
SPERT	1	0
MYOZ1	1	0
LUZP4	1	0
COMMD3-BMI1	1	0
RPS27	1	0
PDLIM1	1	0
MED14	1	0
FXN	1	0
SORBS2	1	0
MLX	1	0
KDM5A	1	0
IMPA2	1	0
HAUS2	1	0

Factor	Degree	Betweenness centrality
PFDN5	1	0
NCOA2	1	0
BAG4	1	0
CDKN1A	1	0
HSF2	1	0
EWSR1	1	0
HSPH1	1	0
RIPK2	1	0
TP53	1	0
DDX24	1	0
SIL1	1	0
RPA2	1	0
TRAF2	1	0
TG	1	0
DPYSL5	1	0
DNAJB9	1	0
DGCR8	1	0
TOPBP1	1	0
PPP4R4	1	0
CUL4B	1	0
HNRNPA2B1	1	0
BCAR1	1	0
SIRT1	1	0
TMEM132A	1	0
EP300	1	0
A2M	1	0
MIR1306	1	0
TUBGCP3	1	0
CALU	1	0
SHMT2	1	0
KCND3	1	0
TMEM62	1	0
LOC100506057	1	0
PRKCZ	1	0
ENO1	1	0
CAMKV	1	0
DYRK1B	1	0
MAP3K9	1	0
POGK	1	0
KLHL13	1	0
ROR2	1	0
TAOK3	1	0
IRAK2	1	0
PRKCG	1	0
ZNF74	1	0
CSNK1E	1	0
IFIT1	1	0
RPS6	1	0

Factor	Degree	Betweenness centrality
FBXO27	1	0
NEK8	1	0
NHLRC1	1	0
KLHL6	1	0
MYLK3	1	0
CAMK4	1	0
FASN	1	0
NEK11	1	0
STARD13	1	0
CYP17A1	1	0
DCLK2	1	0
PRKAA2	1	0
RPS6KA3	1	0
KCNG1	1	0
FBXO24	1	0
IRF2	1	0
PASK	1	0
INSRR	1	0
TRIM17	1	0
STK33	1	0
RPS6KA5	1	0
TRADD	1	0
DTX4	1	0
AARS	1	0
CDK3	1	0
GTF2IRD2	1	0
PRKD1	1	0
MARCH9	1	0
MIR1236	1	0
RGS7	1	0
KLHL15	1	0
RGS9	1	0
DYRK4	1	0
EIF2AK2	1	0
FBXW5	1	0
EEF2	1	0
LATS2	1	0
CAMK2D	1	0
RPL5	1	0
ACVR1C	1	0
TBX22	1	0
DLX6	1	0
NFIC	1	0
HMGA1	1	0
ATF3	1	0
STK32C	1	0
NFKB1	1	0
KLHL26	1	0

Factor	Degree	Betweenness centrality
KLHL29	1	0
FBXL13	1	0
FBXL14	1	0
STAT1	1	0
WHSC1	1	0
VPS41	1	0
CDKN2A	1	0
SLFN11	1	0
TRIM36	1	0
KLHL25	1	0
RIPK1	1	0
STK32B	1	0
FBXW11	1	0
WSB2	1	0
MAP3K6	1	0
MYO3B	1	0
TRIM37	1	0
ZBTB20	1	0
RDBP	1	0
LRSAM1	1	0
TSSK1B	1	0
TNK2	1	0
MKX	1	0
MKNK1	1	0
SNORD21	1	0
EPHA1	1	0
JAK1	1	0
EPHB1	1	0
KHDRBS2	1	0
RGS11	1	0
FOXM1	1	0
MYH9	1	0
KSR2	1	0
PRPF19	1	0
BBX	1	0
RPL7	1	0
PPARD	1	0
GLUL	1	0
ASB9	1	0
EIF2C2	1	0
SAMD3	1	0
ALDH2	1	0
BAK1	1	0
ICT1	1	0
SRRM2	1	0
CASP3	1	0
PCK1	1	0
HIST2H2BE	1	0

Factor	Degree	Betweenness centrality
EIF2C1	1	0
PPP2R1B	1	0
MUC7	1	0
CD14	1	0
CALM1	1	0
CALM3	1	0
CALM2	1	0
UBC	1	0
SP1	1	0
ZDHHC17	1	0
MARK4	1	0
RAB8A	1	0
PRKAB1	1	0
GSTK1	1	0
NXT2	1	0
UCHL5	1	0
SHBG	1	0
PML	1	0
POLE2	1	0
PAX8	1	0
EGLN1	1	0
ENO3	1	0
RELA	1	0
CNR2	1	0
ADRM1	1	0
PSMC1	1	0
WRAP73	1	0
MLF1	1	0
BAG5	1	0
SMARCD2	1	0
HMOX1	1	0
PSMD6	1	0
SGTA	1	0
PTN	1	0
ASB11	1	0
AXIN1	1	0
PRAME	1	0
LRIF1	1	0
PSMC5	1	0
TUBB1	1	0
PSMD13	1	0
PAAF1	1	0
REC8	1	0
PSMC4	1	0
URI1	1	0
PINK1	1	0
MCM5	1	0
DNAJB2	1	0

Factor	Degree	Betweenness centrality
BAG1	1	0
GPN1	1	0
WDR16	1	0
FBXO46	1	0
GPR37	1	0
APEX1	1	0
PPIB	1	0
RNF185	1	0
TXNDC11	1	0
MLLT3	1	0
EIF2B2	1	0
MIPOL1	1	0
MKKS	1	0
STK24	1	0
CDKN2AIP	1	0
SNORD55	1	0
CAV1	1	0
UBE2S	1	0
ASPSCR1	1	0
RRP12	1	0
CDC25A	1	0
ZFAND2B	1	0
RSU1	1	0
RPS13	1	0
CTNNBL1	1	0
RNF19A	1	0
RPS8	1	0
RPL23	1	0
FANCI	1	0
RPS25	1	0
SMARCA5	1	0
AR	1	0
PHKG2	1	0
C11orf82	1	0
NGLY1	1	0
PPP3CA	1	0
STMN1	1	0
NUPL1	1	0
AVPR2	1	0
H2AFJ	1	0
BTRC	1	0
PDXDC1	1	0
NEK2	1	0
SNORD14B	1	0
TRIM21	1	0
UBXN11	1	0
RNF8	1	0
CHEK2	1	0

Factor	Degree	Betweenness centrality
PLK1	1	0
TOM1L1	1	0
CNOT10	1	0
TRIP12	1	0
BRSK2	1	0
ESPL1	1	0
CEP19	1	0
TTK	1	0
WAPAL	1	0
RPL24	1	0
BRAT1	1	0
BSG	1	0
SYMPK	1	0
NCAPH	1	0
SPC24	1	0
CASP7	1	0
TBC1D10B	1	0
CEP55	1	0
DGCR6	1	0
UBB	1	0
FKBP15	1	0
TRIM13	1	0
GIGYF2	1	0
DIAPH3	1	0
HELLS	1	0
STAG2	1	0
L3MBTL1	1	0
KIAA1524	1	0
POLR3C	1	0
SNORD38B	1	0
YWHAH	1	0
LMNB2	1	0
FAM104A	1	0
BCCIP	1	0
YWHAG	1	0
UBXN10	1	0
LOC100508408	1	0
CDK1	1	0
COG4	1	0
MAPK8IP2	1	0
BAIAP2L1	1	0
HOOK1	1	0
LYAR	1	0
BID	1	0
SNORA21	1	0
LMNA	1	0
SPA17	1	0
DNM2	1	0

Factor	Degree	Betweenness centrality
ANXA1	1	0
ASB7	1	0
PRPF40A	1	0
PRRC2A	1	0
TIMP2	1	0
CCNA1	1	0
ATP4A	1	0
ABL1	1	0
ACTN3	1	0
PARP1	1	0
ADRA2A	1	0
ADRBK1	1	0
ALDOA	1	0
AMHR2	1	0
ANXA2	1	0
APOA1	1	0
ARF6	1	0
ASGR1	1	0
BAX	1	0
BCR	1	0
PRDM1	1	0
BMPR1A	1	0
BMX	1	0
FMNL1	1	0
CACNA1A	1	0
CAMK2G	1	0
CASP9	1	0
CCNB1	1	0
CCNH	1	0
CHM	1	0
CLCN2	1	0
CP	1	0
CSF1R	1	0
CSNK1A1	1	0
CSNK2A2	1	0
CSRP1	1	0
CTNNB1	1	0
DAG1	1	0
DMPK	1	0
DMWD	1	0
DNMT1	1	0
DUSP9	1	0
EEF1A2	1	0
EPHA2	1	0
EIF4EBP1	1	0
ENO2	1	0
ERG	1	0
F7	1	0

Factor	Degree	Betweenness centrality
FANCC	1	0
PTK2B	1	0
FARSA	1	0
FDXR	1	0
FGFR3	1	0
FGR	1	0
FHIT	1	0
MTOR	1	0
FYN	1	0
XRCC6	1	0
GAPDH	1	0
GCGR	1	0
GJA1	1	0
GRK4	1	0
GRK5	1	0
NR3C1	1	0
HCK	1	0
HLA-C	1	0
NR4A1	1	0
HSPA1A	1	0
HSPA1B	1	0
HSPA4	1	0
ICAM1	1	0
ID2	1	0
ILK	1	0
ITPR1	1	0
KCNA5	1	0
KPNA2	1	0
RPSA	1	0
LYZ	1	0
SMAD3	1	0
MAGEA11	1	0
MAP2	1	0
MAX	1	0
MDH1	1	0
MEOX2	1	0
MPG	1	0
MSH4	1	0
MTHFD1	1	0
NCL	1	0
NME2	1	0
NTRK1	1	0
NTRK2	1	0
NTRK3	1	0
DDR2	1	0
PAFAH1B2	1	0
CDK18	1	0
CDK14	1	0

Factor	Degree	Betweenness centrality
PGK1	1	0
SERPINA1	1	0
PIK3C2B	1	0
PIM1	1	0
PLCG2	1	0
PLEC	1	0
PPP2CA	1	0
PPP2R1A	1	0
PPP5C	1	0
PRKACB	1	0
PRKAR1A	1	0
PRKCA	1	0
PRKCH	1	0
PKN2	1	0
PRKCSH	1	0
MAPK4	1	0
MAPK6	1	0
MAP2K1	1	0
PSMA1	1	0
PSMC2	1	0
PSMC3	1	0
PSMD3	1	0
PTGS2	1	0
PTPRC	1	0
PYGB	1	0
RAD52	1	0
RAP1A	1	0
RCN2	1	0
RFC3	1	0
SNORA62	1	0
RPL6	1	0
RPL18A	1	0
RPL22	1	0
RPLP0	1	0
RPN1	1	0
RPS6KA2	1	0
RPS6KB1	1	0
RPS11	1	0
SKP2	1	0
SLC2A4	1	0
SMARCC1	1	0
SRPK1	1	0
STAU1	1	0
STK11	1	0
AURKC	1	0
TAF10	1	0
TIE1	1	0
TJP1	1	0

Factor	Degree	Betweenness centrality
TNFRSF1A	1	0
TP53BP1	1	0
TSSC1	1	0
TTC1	1	0
DNAJC7	1	0
TTN	1	0
UFD1L	1	0
VBP1	1	0
ZNF215	1	0
ZYX	1	0
BAG6	1	0
PDHX	1	0
DGCR14	1	0
BAP1	1	0
DYRK2	1	0
TEAD2	1	0
FKBP6	1	0
RAE1	1	0
PPFIBP1	1	0
PIK3R3	1	0
COPS3	1	0
RGS20	1	0
CDK13	1	0
IQGAP1	1	0
KAT2B	1	0
CDK5R1	1	0
AP3D1	1	0
AIP	1	0
UNC119	1	0
RQCD1	1	0
KCNQ4	1	0
CBFA2T2	1	0
NUMBL	1	0
EIF2AK3	1	0
UBE3C	1	0
RNF40	1	0
GIT2	1	0
RHOBTB1	1	0
PARP2	1	0
DNAJB6	1	0
PSMD14	1	0
TUBB4A	1	0
TUBB4B	1	0
HAX1	1	0
MERTK	1	0
TRIM38	1	0
NXF1	1	0
CCT4	1	0

Factor	Degree	Betweenness centrality
CAMKK2	1	0
GNA13	1	0
SDCCAG3	1	0
TUBGCP2	1	0
FAF1	1	0
IRAK3	1	0
COPE	1	0
ICK	1	0
SCMH1	1	0
SPG20	1	0
TAB2	1	0
MAST2	1	0
ANKLE2	1	0
MDN1	1	0
ZC3H7B	1	0
TRIM2	1	0
UBR4	1	0
TSSK2	1	0
NUP62	1	0
HSPBP1	1	0
FKBP8	1	0
PRPF6	1	0
ARIH1	1	0
POT1	1	0
CLIC4	1	0
UBXN7	1	0
GGA1	1	0
WIPI2	1	0
FBXO9	1	0
FAM162A	1	0
SRPK3	1	0
EIF2AK1	1	0
ERLEC1	1	0
BZW2	1	0
ANAPC2	1	0
MED31	1	0
DERL2	1	0
DYNC1LI1	1	0
TFDP3	1	0
ANAPC7	1	0
JKAMP	1	0
NUB1	1	0
INPP5K	1	0
ROPN1	1	0
DET1	1	0
MED9	1	0
HEATR1	1	0
RFWD3	1	0

Factor	Degree	Betweenness centrality
RIF1	1	0
RCBTB1	1	0
VPS53	1	0
STYK1	1	0
LGR4	1	0
G2E3	1	0
RNF126	1	0
SELS	1	0
PARD3	1	0
ANKMY2	1	0
NUP107	1	0
C12orf10	1	0
C12orf44	1	0
FKBPL	1	0
DMRTA1	1	0
INF2	1	0
CLEC7A	1	0
CENPH	1	0
ACD	1	0
CDK15	1	0
TAF1D	1	0
FBXL15	1	0
MAP7D3	1	0
ASB13	1	0
UBA5	1	0
CCDC134	1	0
HDAC11	1	0
FBXL18	1	0
TRIM8	1	0
NUAK2	1	0
TRIM56	1	0
ROPN1L	1	0
C1orf124	1	0
LZTS2	1	0
SLX4	1	0
DGAT2	1	0
KLHL22	1	0
SPSB3	1	0
TMEM67	1	0
NLRP12	1	0
METTL18	1	0
IGSF8	1	0
FOXP2	1	0
EGLN3	1	0
WBSCR22	1	0
SSX2IP	1	0
HSF5	1	0
ASB17	1	0

Factor	Degree	Betweenness centrality
RNF19B	1	0
UBLCP1	1	0
HUS1B	1	0
RAB40A	1	0
CCDC117	1	0
METTL21A	1	0
ROPN1B	1	0
SH3RF2	1	0
FSIP1	1	0
DCP2	1	0
KCTD6	1	0
METTL20	1	0
KLHL34	1	0
KLHL10	1	0
KLHL38	1	0
USP50	1	0
TRIM73	1	0
TRIM74	1	0
FSIP2	1	0
SNORA6	1	0
SNORA8	1	0
SNORA1	1	0
SNORA18	1	0
SNORA40	1	0
SNORA32	1	0
SNORD5	1	0
MIR1304	1	0
LOC100507353	1	0

Supplementary Table S6. Hemoglobin levels in follicular fluid samples.

FF sample	Hb before processing (g/dL)	Hb after processing (g/dL)
1	0.02 (0.00)	0.00 (0.00)
2	0.02 (0.00)	0.00 (0.00)
3	0.00 (0.00)	0.00 (0.00)
4	0.02 (0.00)	0.00 (0.00)
5	0.02 (0.00)	0.00 (0.00)
6	0.01 (0.00)	0.00 (0.00)
7	0.04 (0.00)	0.00 (0.00)
8	0.10 (0.00)	0.00 (0.00)
9	0.00 (0.00)	0.00 (0.00)
10	0.00 (0.00)	0.00 (0.00)
11	0.00 (0.00)	0.00 (0.00)
12	0.03 (0.00)	0.00 (0.00)
13	0.02 (0.00)	0.00 (0.00)
14	0.02 (0.00)	0.00 (0.00)
15	0.06 (0.00)	0.00 (0.00)
16	0.03 (0.00)	0.00 (0.00)
17	0.02 (0.00)	0.00 (0.00)
18	0.02 (0.00)	0.00 (0.00)
19	0.08 (0.00)	0.00 (0.00)
20	0.00 (0.00)	0.00 (0.00)
21	0.02 (0.00)	0.00 (0.00)
22	0.00 (0.00)	0.00 (0.00)
23	0.05 (0.00)	0.00 (0.00)
24	0.00 (0.00)	0.00 (0.00)
25	0.04 (0.00)	0.00 (0.00)
26	0.00 (0.00)	0.00 (0.00)

FF, follicular fluid; Hb, hemoglobin; measurements were performed in duplicate and results are represented as mean (SD).
